# Non-peptide compounds from *Kronopolites svenhedini* (Verhoeff) and their antitumor and iNOS inhibitory activities

**DOI:** 10.3762/bjoc.19.59

**Published:** 2023-06-07

**Authors:** Yuan-Nan Yuan, Jin-Qiang Li, Hong-Bin Fang, Shao-Jun Xing, Yong-Ming Yan, Yong-Xian Cheng

**Affiliations:** 1 School of Pharmacy, Guangdong Pharmaceutical University, Guangzhou 510006, PR China; 2 Institute for Inheritance-Based Innovation of Chinese Medicine, School of Pharmaceutical Sciences, Health Science Center, Shenzhen University, Shenzhen 518060, PR China; 3 Department of Pathogen Biology, Health Science Center, Shenzhen University, Shenzhen 518060, PR China

**Keywords:** arthropod, iNOS, *Kronopolites svenhedini* (Verhoeff), non-peptide small molecules

## Abstract

Six new compounds, including a tetralone **1**, two xanthones **2** and **3**, a flavan derivative **4**, and two nor-diterpenoids **7** and **8**, accompanied by two known flavan derivatives **5** and **6** and a known olefine acid (**9**) were isolated from whole bodies of *Kronopolites svenhedini* (Verhoeff). The structures of the new compounds were determined by 1D and 2D nuclear magnetic resonance (NMR) and other spectroscopic methods, as well as computational methods. Selected compounds were evaluated for their biological properties against a mouse pancreatic cancer cell line and inhibitory effects on iNOS and COX-2 in RAW264.7 cells.

## Introduction

The millipede (class Diplopoda) is a pervasive arthropod in nature, functioning as a decomposer in forest ecosystems [1]. Most current research on millipedes centers around biological sciences and environmental sciences, such as community changes [2–4]. Limited studies on millipede chemical composition and biological activity have revealed the presence of antimicrobial peptides [5], defensive alkaloids [6], and defensive long chain alcohol acetates [7]. Historically, in China, numerous records documented the utilization of animals like arthropods for medicinal purposes. Millipedes hereby represent a traditional Chinese medicine (TCM) with anti-inflammatory, analgesic, stomach-soothing, and fatigue-relieving effects [8]. *K. svenhedini* (Diplopoda, Strongylosomidae) is a millipede species first described by Verhoeff in 1934 [9]. Based on literature [10], we know that *K. svenhedini* is the animal source of the TCM millipedes. In our studies of arthropods over the years, we have found that non-peptide small molecules play a significant role in chemical structures and biological activities [11–16].

**Figure 1:**
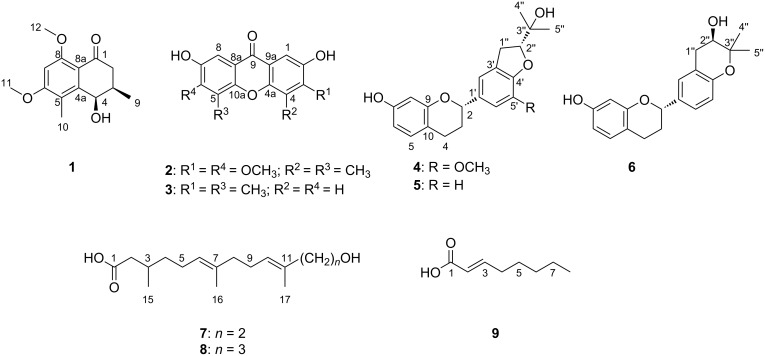
Structures of compounds **1**–**9**.

In examining the chemical constituents of the millipede *K. svenhedini*, the focus was directed toward non-peptide small molecules, leading to the isolation of six new and three known compounds (Figure 1) from its extract. These structures were determined by 1D and 2D NMR spectra and the experimental and calculated electronic circular dichroism (ECD) spectra. The six new compounds have been named kronopoone A (**1**), kronopoiols A (**2**) and B (**3**), 5-*O*-methyldaphnegiralin C_1_ (**4**), and kronoponoids A (**7**) and B (**8**). Biological activity experiments were conducted with the isolated compounds, revealing that compounds **3**–**5** exhibited the expression of iNOS in a dose-dependent manner.

## Results and Discussion

### Structural identification

**Figure 2:**
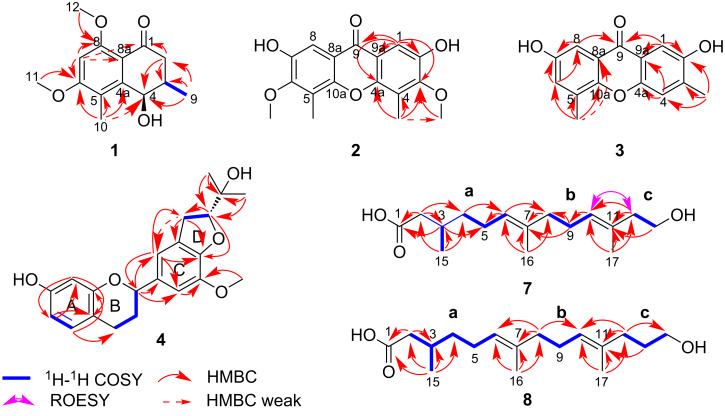
Key ^1^H–^1^H COSY, HMBC, and ROESY correlations of compounds **1**–**4**, **7**, and **8**.

**Figure 3:**
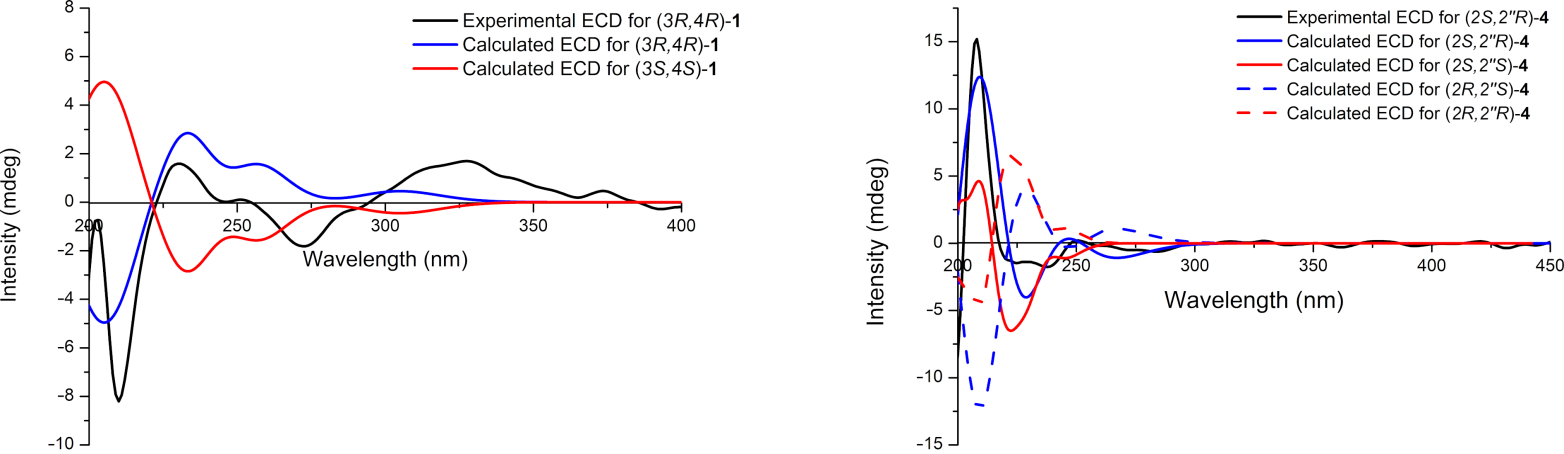
Calculated and experimental ECD spectra of compounds **1** and **4**.

Compound **1**, a yellow gum, possesses the molecular formula C_14_H_18_O_4_ (six degrees of unsaturation), as deduced from its HRESIMS [M + H]^+^ ion peak at *m*/*z* 251.1274 (calcd for C_14_H_19_O_4_, 251.1278). The ^1^H NMR data (Table 1 and Figure S1 in Supporting Information File 1) display one aromatic proton [δ_H_ 7.02 (s, 1H, H-7)], two methoxy signals [δ_H_ 3.96 (s, 3H, H_3_-12) and 3.73 (s, 3H, H_3_-11)], and two methyl signals [δ_H_ 2.51 (s, 3H, H_3_-10) and 1.09 (d, *J* = 6.8 Hz, 3H, H_3_-9)]. The ^13^C NMR and DEPT spectra of compound **1** (Table 1 and Figure S2 in Supporting Information File 1) exhibit 14 resonances attributable to two methyl groups, two methoxy carbons, one methylene, three methines (one sp^2^ and one of them oxygenated), one ketone, and five sp^2^ carbons (two of them oxygenated). Some of these signals resemble those of 8-*O*-methylteratosphaerone B [17], suggesting compound **1** being an analogue, but with an additional methyl group on the benzene ring. The HMBC correlations (Figure 2 and Figure S5 in Supporting Information File 1) of H_3_-10/C-4 (δ_C_ 72.9, weak), C-4a (δ_C_ 136.1), C-5 (δ_C_ 124.5), C-6 (δ_C_ 148.5) disclosed that C-10 is connected to C-5 in compound **1**. The coupling constant was used to determine the relative configuration of the cyclohexanone segment in **1**. The small coupling constant (*J*_3,4_ = 3.0 Hz) indicated that H-3 and H-4 are on the same side of the ring, corroborated by the literature [18]. The absolute configuration of compound **1** was identified as 3*R*,4*R* in accordance with the experimental and calculated ECD spectra (Figure 3 and Figure S7 in Supporting Information File 1). As a result, the structure of **1** was defined and designated as kronopoone A.

**Table 1:** ^1^H (600 MHz) and ^13^C NMR (150 MHz) data of compound **1** (δ in ppm, *J* in Hz, methanol-*d*_4_).

No.	δ_H_ (mult, *J*, amount)	δ_C_ mult	No.	δ_H_ (mult, *J*, amount)	δ_C_ mult

C-1		201.3 C	C-7	7.02 (s, 1H)	110.8 CH
C-2	2.64 (dd, *J* = 17.2, 10.3, 1H) 2.48 (dd, *J* = 17.2, 4.7, 1H)	43.7 CH_2_	C-8		158.0 C
C-3	2.36 (m, 1H)	35.8 CH	C-8a		145.6 C
C-4	4.69 (d, *J* = 3.0, 1H)	72.9 CH	C-9	1.09 (d, *J* = 6.8, 3H)	16.3 CH_3_
C-4a		136.1 C	C-10	2.51 (s, 3H)	14.1 CH_3_
C-5		124.5 C	C-11	3.73 (s, 3H)	60.7 CH_3_
C-6		148.5 C	C-12	3.96 (s, 3H)	56.3 CH_3_

Compound **2**, isolated as a brown solid, was showed to have the molecular formula C_17_H_16_O_6_ (ten degrees of unsaturation) on the basis of its HRESIMS [M + H]^+^ ion peak at *m*/*z* 317.1008 (calcd for C_17_H_17_O_6_, 317.1020). The ^1^H NMR data (Table 2 and Figure S8 in Supporting Information File 1) reveal an aromatic signal [δ_H_ 7.47 (s, 2H, H-1, H-8)], a methoxy signal [δ_H_ 3.95 (s, 6H, H_3_-11, H_3_-14)], and a methyl signal [δ_H_ 2.48 (s, 6H, H_3_-12, H_3_-13)]. The ^13^C NMR and DEPT spectra (Table 2 and Figure S9 in Supporting Information File 1) show that compound **2** is comprised of 9 carbons, including one methyl, one methoxy carbon, one sp^2^ methine, one ketone, and five sp^2^ carbons (three of them oxygenated). The ^1^H and ^13^C NMR data and the molecular formula indicated that the compound possesses the same two pentasubstituted benzene rings, suggesting an axially symmetric structure. The methoxy group is situated at C-3, as determined by the HMBC correlation (Figure 2 and Figure S12 in Supporting Information File 1) of H_3_-11/C-3 (δ_C_ 154.1). The hydroxy group and the methyl group are positioned at C-2 and C-4, respectively, as deduced by comparing the HMBC correlations of H-1/C-2 (δ_C_ 148.7), C-9a (δ_C_ 118.1), C-9 (δ_C_ 178.5), and H_3_-12/C-3, C-4 (δ_C_ 121.6), C-4a (δ_C_ 151.0), C-11 (δ_C_ 61.0, weak) with those in the literature [18–21]. These results suggest that the second benzene ring shares the same structure. By comparing the ^1^H and ^13^C chemical shifts with similar compounds [19–22], the NMR data implied the presence of C-8a–C-9–C-9a and C-4a–O–C-10a bonds in the structure of **2**. Consequently, the structure of compound **2** was identified and named kronopoiol A.

**Table 2:** ^1^H (600 MHz) and ^13^C NMR (150 MHz) data of compounds **2** and **3** (δ in ppm, *J* in Hz, methanol-*d*_4_).

No.	**2**			**3**	
	
	δ_H_ (mult, *J*, amount)	δ_C_ mult		δ_H_ (mult, J, amount)	δ_C_ mult
	
C-1	7.47 (s, 1H, overlap)	108.3 CH		7.47 (s, 1H)	108.2 CH
C-2		148.7 C			153.8 C
C-3		154.1 C			137.5 C
C-4		121.6 C		7.39 (d, *J* = 1.0, 1H)	120.5 CH
C-4a		151.0 C			151.6 C
C-5		121.6 C			130.2 C
C-6		154.1 C		7.16 (dd, *J* = 3.1, 1.0, 1H)	126.1 CH
C-7		148.7 C			154.4 C
C-8	7.47 (s, 1H, overlap)	108.3 CH		7.38 (d, *J* = 3.1, 1H)	107.2 CH
C-8a		118.1 C			122.5 C
C-9		178.5 C			179.1 C
C-9a		118.1 C			120.5 CH
C-10a		151.0 C			150.1 C
C-11	3.95 (s, 3H, overlap)	61.0 CH_3_		2.37 (s, 3H)	17.1 CH_3_
C-12	2.48 (s, 3H, overlap)	9.2 CH_3_		2.52 (s, 3H)	15.8 CH_3_
C-13	2.48 (s, 3H, overlap)	9.2 CH_3_			
C-14	3.95 (s, 3H, overlap)	61.0 CH_3_			

Compound **3**, a brown solid, was assigned the molecular formula C_15_H_12_O_4_ (ten degrees of unsaturation), by analysis of its HRESIMS [M + H]^+^ ion peak at *m*/*z* 257.0802 (calcd for C_15_H_13_O_4_, 257.0808). The ^1^H NMR data (Table 2 and Figure S14 in Supporting Information File 1) show a typical AB spin system [δ_H_ 7.47 (s, 1H, H-1), 7.39 (d, *J* = 1.0 Hz, 1H, H-4)], which corresponds to a 1,2,4,5-tetrasubstituted benzene substructure. Additional aromatic proton signals [δ_H_ 7.38 (d, *J* = 3.1 Hz, 1H, H-8) and δ_H_ 7.16 (dd, *J* = 3.4, 1.0 Hz, 1H, H-6)] suggest the presence of a 1,2,3,5-tetrasubstituted benzene substructure. The ^13^C NMR and DEPT spectra of **3** (Table 2 and Figure S15 in Supporting Information File 1) exhibit 15 resonances classified into two methyls, four sp^2^ methines, one ketone, and eight sp^2^ carbons (four of them oxygenated). The two methyl groups are positioned at C-3 and C-5, as deduced by the HMBC correlations (Figure 2 and Figure S18 in Supporting Information File 1) of H_3_-11/C-2 (δ_C_ 153.8), C-3 (δ_C_ 137.5), C-4 (δ_C_ 120.5) and H_3_-12/C-5 (δ_C_ 130.2), C-6 (δ_C_ 126.1), C-10a (δ_C_ 150.1), C-8a (δ_C_ 61.0, weak). By comparing the HMBC correlations of H-1/C-2, C-9a (δ_C_ 120.5), C-9 (δ_C_ 179.1), and H-8/C-7 (δ_C_ 154.4), C-9 (δ_C_ 179.1) with those in the literature [18–22], it was concluded that the hydroxy groups are positioned at C-2 and C-7, respectively. A comparison between compounds **2** and **3** revealed that both possess C-8a–C-9–C-9a and C-4a–O–C-10a bonds. As a result, the structure of **3** is defined and named as kronopoiol B.

Compound **4** was obtained as a brown solid. The molecular formula of it was determined to be C_21_H_24_O_5_ (ten degrees of unsaturation) deduced from its HRESIMS [M + H]^+^ ion peak at *m*/*z* 357.1680 (calcd for C_21_H_25_O_5_, 357.1697). The ^1^H NMR data (Table 3 and Figure S20 in Supporting Information File 1) show three typical aromatic signals [δ_H_ 6.86 (m, 1H, H-5, overlap), 6.31 (dd, *J* = 8.2, 2.4 Hz, 1H, H-6), and 6.26 (d, *J* = 2.4 Hz, 1H, H-8)], suggesting the presence of a 1,2,4-trisubstituted benzene substructure. Additionally, two aromatic signals at δ_H_ 6.86 (m, 1H, H-2', overlap) and δ_H_ 6.84 (s, 1H, H-6') are observed in the ^1^H NMR spectrum, indicating the presence of a 1,3,4,5-tetrasubstituted benzene substructure. The ^13^C NMR and DEPT spectra of **4** (Table 3 and Figure S21 in Supporting Information File 1) contain 21 resonances ascribed to two methyls, one methoxy carbon, three methylenes, seven methines (five sp^2^ and two of them oxygenated), one oxygenated carbon, and seven sp^2^ carbons (four of them oxygenated). Based on this information, compound **4** was deduced to be similar to daphnegiralin C_1_ [23], with both sharing the same 7-hydroxyflavan skeleton. The distinction in compound **4** is an additional methoxy group, which is connected to C-5' as supported by the HMBC correlation (Figure 2 and Figure S24 in Supporting Information File 1) of 5'-OCH_3_ (δ_H_ 3.85)/C-5' (δ_C_ 136.1). Two asymmetric carbon centers are present at C-2 and C-2″ in compound **4**. According to the literature [22], the absolute configuration at C-2 for **4** was assigned as *S*, from the Cotton effects in its ECD curve (Figure S26 in Supporting Information File 1) [283 nm (Δε −0.71)]. The absolute configuration of **4** was determined as *2S,2″R* based on the comparison of the experimental and ECD spectra (Figure 3 and Figure S26 in Supporting Information File 1). Consequently, the structure of **4** is defined and named 5-*O*-methyldaphnegiralin C_1_.

**Table 3:** ^1^H (500 MHz) and ^13^C NMR (150 MHz) data of **4** (δ in ppm, *J* in Hz, methanol-*d*_4_).

No.	δ_H_ (mult, *J*, amount)	δ_C_ mult	No.	δ_H_ (mult, *J*, amount)	δ_C_ mult

C-2	4.92 (m, 1H)	79.2 CH	C-3'		129.8 C
C-3	2.12 (m, 1H) 2.00 (m, 1H)	31.6 CH_2_	C-4'		149.2 C
C-4	2.86 (ddd, *J* = 16.5, 11.3, 5.7, 1H) 2.67 (m, 1H)	25.5 CH_3_	C-5'		145.2 CH
C-5	6.86 (m, 1H, overlap)	131.0 CH	C-6'	6.84 (s, 1H)	111.4 CH
C-6	6.31 (dd, *J* = 8.2, 2.4, 1H)	109.1 CH	C-1''	3.19 (m, 2H)	32.0 CH_2_
C-7		157.6 C	C-2''	4.63 (t, *J* = 9.0, 1H)	91.1 CH
C-8	6.26 (d, *J* = 2.4, 1H)	104.1 CH	C-3''		72.5 C
C-9		157.1 C	C-4''	1.24 (s, 3H)	25.5 CH_3_
C-10		114.3 C	C-5''	1.26 (s, 3H)	25.0 CH_3_
C-1'		136.5 C	5'-OCH_3_	3.85 (s, 3H)	56.8 CH_3_
C-2'	6.86 (m, 1H, overlap)	116.2 CH			

Compound **7**, a light yellow gum, has the molecular formula C_16_H_28_O_3_ (three degrees of unsaturation) as determined by its HRESIMS [M + H]^+^ ion peak at *m*/*z* 269.2116 (calcd for C_16_H_29_O_3_, 269.2111). The ^1^H NMR spectrum (Table 4 and Figure S27 in Supporting Information File 1) displays two olefinic protons [δ_H_ 5.18 (t, *J* = 7.0 Hz, 1H, H-10) and 5.12 (t, *J* = 7.0 Hz, 1H,H-6)], one oxymethylene group [δ_H_ 3.59 (t, *J* = 7.1 Hz, 2H, H_2_-13)], and three methyl signals [δ_H_ 1.63 (s, 3H, H_3_-16), 1.61 (s, 3H, H_3_-15), and 0.96 (s, 3H, H_3_-14)]. The ^13^C NMR and DEPT spectra of compound **7** (Table 4 and Figure S28 in Supporting Information File 1) contain 16 resonances attributable to three methyls, seven methylenes (one of them oxygenated), three methines (two sp^2^), one carbonyl carbon, and two sp^2^ quaternary carbons. The ^1^H–^1^H COSY spectrum (Figure 2 and Figure S29 in Supporting Information File 1) of compound **7** disclosed the existence of correlations of H_2_-2 (δ 2.29, 2.08)/H-3 (δ_H_ 1.91, 1.93)/H_2_-4 (δ_H_ 1.38, 1.24)/H_2_-5 (δ_H_ 2.03, 2H)/H-6 (δ_H_ 5.12), H-3 (δ_H_ 1.93)/H_2_-15 (δ_H_ 0.96), H_2_-8 (δ_H_ 2.03, 2H)/H_2_-9 (δ_H_ 2.11, 2H)/H-10 (δ_H_ 5.18), and H_2_-12 (δ_H_ 2.20, 2H)/H_2_-13 (δ_H_ 3.59, 2H), which revealed three partial structures **a** (C-2 to C-6), **b** (C-8 to C-10), and **c** (C-12 to C-13). The partial structures **a** and **b** were connected to C-7 by the correlations of H_3_-16 (δ_H_ 1.61)/C-6 (δ_C_ 125.6), C-7 (δ_C_ 136.0), C-8 (δ_C_ 40.7), and H-8/C-6, C-7 in the HMBC spectrum (Figure 2 and Figure S31 in Supporting Information File 1). The partial structures **b** and **c** were connected to C-11, as confirmed by the HMBC correlations of H_3_-17 (δ_H_ 1.63)/C-10 (δ_C_ 127.4), C-11 (δ_C_ 132.9), C-12 (δ_C_ 43.8), and H-12/C-10, C-11. The presence of a conjugated carboxylic acid was verified by the HMBC correlation of H_2_-2 to C-1 (δ_C_ 177.1). Concerning the geometry of **7**, the ROESY correlation (Figure 2 and Figure S32 in Supporting Information File 1) of H-10/H_2_-12 disclosed that the ∆^10,11^ configuration was *E*. However, the overlapping signals of H_2_-5 and H_2_-8 made it difficult to determine the geometry of the ∆^6,7^ in the same manner. By comparing the ^1^H and ^13^C chemical shifts with those of similar compounds [24–28], the geometry of the ∆^6,7^ was determined to be *E*. The configuration at C-3 remains undetermined due to its long carbon chain. As a consequence, the structure of compound **7**, named kronoponoid A, was determined to be as showed in Figure 1.

**Table 4:** ^1^H (500 MHz) and ^13^C NMR (150 MHz) data of compounds **7** and **8** (δ in ppm, *J* in Hz, methanol-*d*_4_).

No.	**7**			**8**	
	
	δ_H_ (mult, *J*, amount)	δ_C_ mult		δ_H_ (mult, *J*, amount)	δ_C_ mult
	
C-1		177.1 C			182.5 C
C-2	2.29 (dd, *J* = 14.8, 6.0, 1H) 2.08 (m, 1H)	42.6 CH_2_		2.19 (m, 1H) 1.94 (m, 1H, overlap)	47.3 CH_2_
C-3	1.93 (m, 1H)	31.1 CH		1.94 (m, 1H, overlap)	32.2 CH
C-4	1.38 (m, 1H) 1.24 (m, 1H)	37.8 CH_2_		1.38 (m, 1H) 1.19 (m, 1H)	38.5 CH_2_
C-5	2.03 (m, 2H, overlap)	26.3 CH_2_		2.02 (m, 1H) 1.98 (m, 1H, overlap)	26.6 CH_2_
C-6	5.12 (t, *J* = 7.0, 1H)	125.6 C		5.16 (m, 1H, overlap)	126.0 CH
C-7		136.0 C			135.6 C
C-8	2.03 (m, 2H, overlap)	40.7 CH_2_		1.98 (m, 2H, overlap)	40.9 CH_2_
C-9	2.11 (m, 2H, overlap)	27.6 CH_2_		2.08 (m, 2H, overlap)	27.9 CH_2_
C-10	5.18 (t, *J* = 7.0, 1H)	127.4 C		5.16 (m, 1H, overlap)	126.0 CH
C-11		132.9 C			135.2 C
C-12	2.20 (m, 2H, overlap)	43.8 CH_2_		2.08 (m, 2H, overlap)	36.8 CH_2_
C-13	3.59 (t, *J* = 7.1, 2H)	61.9 CH_2_		1.75 (m, 2H)	29.0 CH_2_
C-14				3.97 (t, *J* = 6.6, 2H)	68.9 CH_2_
C-15	0.96 (s, 3H)	20.0 CH_3_		0.94 (d, *J* = 6.2, 3H)	20.3 CH_3_
C-16	1.61 (s, 3H)	16.0 CH_3_		1.61 (s, 3H, overlap)	16.1 CH_3_
C-17	1.63 (s, 3H)	16.3 CH_3_		1.61 (s, 3H, overlap)	16.0 CH_3_

Compound **8**, a light yellow gum, possesses the molecular formula C_17_H_30_O_3_ (three degrees of unsaturation) deduced from its HRESIMS [M + H]^+^ ion peak at *m*/*z* 283.2268 (calcd for C_17_H_31_O_3_, 283.2268). The ^1^H NMR data (Table 4 and Figure S34 in Supporting Information File 1) display two olefinic protons [δ_H_ 5.16 (m, 2H, H-6, H-10)], one oxymethylene group [δ_H_ 3.97 (t, *J* = 6.6 Hz, 2H, H_2_-14)], and three methyl signals [δ_H_ 1.61 (s, 6H, H_3_-16, H_3_-17), and 0.94 (d, *J* = 6.2 Hz, 3H, H_3_-15)]. The ^13^C NMR and DEPT spectra (Table 4 and Figure S35 in Supporting Information File 1) show that this substance contains 17 resonances, including three methyls, eight methylenes (one of them oxygenated), three methines (two sp^2^), one carbonyl carbon, and two sp^2^ quaternary carbons. A comparison of the NMR data of compound **8** (Table 4) with that of compound **7**, indicated that both compounds possess the same general skeleton structure, with the only difference being an additional methine group in compound **8**. The ^1^H–^1^H COSY correlations (Figure 2 and Figure S36 in Supporting Information File 1) of H_2_-12 (δ_H_ 1.98, 2H)/H_2_-13 (δ_H_ 2.08, 2H)/H_2_-14 (δ_H_ 3.97, 2H) and the HMBC correlations (Figure 2 and Figure S38 in Supporting Information File 1) of H-12/C-10 (δ_C_ 126.0), C-11 (δ_C_ 135.2), and H-13/C-11 revealed the structure of C-12 to C-14, which differs from **7**. Owing to the overlapping signals between H_2_-5 and H_2_-8, H_2_-9 and H_2_-12, the geometry of the double bonds was determined as 6*E*,10*E* through a comparison of the ^1^H and ^13^C chemical shifts with similar compounds [24–28]. The configuration at C-3 remains undetermined due to its long carbon chain. Thus far, the structure of compound **8** was identified as shown in Figure 1 and named as kronoponoid B.

Of note, the structures of compounds **2**–**4** are common in plants but rare in animals. Whether these compounds originate from plants or animals so far remains unknown.

In addition to the above mentioned compounds, three known compounds were identified as daphnegiralin C_1_ (**5**) [23], daphnegiranol C_1_ (**6**) [29], and (*E*)-oct-2-enoic acid (**9**) [30] by comparing their spectroscopic data with those in the literature.

### Biological evaluation

**Figure 4:**
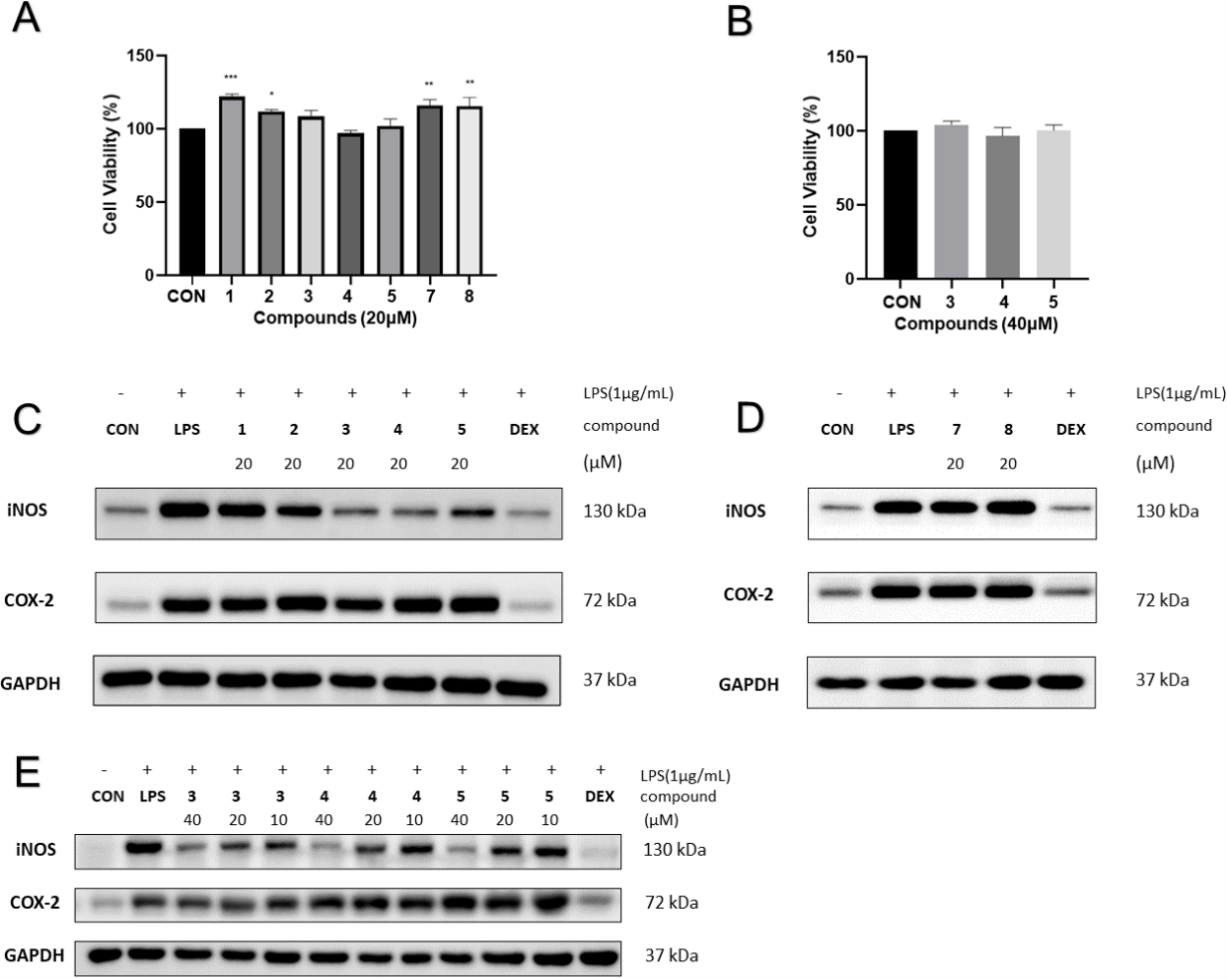
Biological evaluation. Compounds **3**‒**5** inhibited the expression of LPS-induced pro-inflammatory expression of iNOS in RAW264.7 cells. Cells were treated with corresponding concentrations of compounds or DMSO for 2 h before being exposed to 1 μg/mL LPS for 12 h. (A) and (B), the proliferation of RAW264.7 cells in response to compounds at 20 and 40 μM was assessed by the CCK-8 assay. Data represent mean ± SEM values of three experiments. *p < 0.05, ** *p* < 0.01, and *** *p* < 0.001 compared with the CON group (DMSO alone). (C)‒(E), Western blotting was used to determine the protein levels of iNOS and COX-2, using GAPDH as a control and dexamethasone (DEX) as a positive reference drug.

To explore the bioactive potential of the isolated compounds, cytotoxic and anti-inflammatory properties were evaluated. In particular, a mouse pancreatic cancer cell line (Panc02-h7-GP-GFP) was used to determine cytotoxicity. Additionally, LPS-induced pro-inflammatory expression of iNOS and COX-2 in RAW264.7 cells was evaluated. Antitumor activity of compounds **1**–**5**, **7**, and **8** was assessed via a cell proliferation assay using Panc02-h7-GP-GFP cells. Unfortunately, none of the compounds did inhibit the proliferation of Panc02-h7-GP-GFP cells at a concentration of 20 μM (Figure S41 in Supporting Information File 1). On the other hand, an enhancement of CD8^+^ T cells was investigated at corresponding concentrations of compounds **2**–**5**. Regrettably, no enhancement of CD8^+^ T cells was observed (Figure S43 in Supporting Information File 1). To examine the toxicity of compounds **1**–**5**, **7**, and **8**, the CCK-8 assay was employed to detect the viability of RAW264.7 cells. The results indicate that the compounds did not exhibit significant toxicity toward RAW264.7 cells at the utilized concentrations (Figure 4A and B). Meanwhile, compounds **1**‒**5**, **7**, and **8** were evaluated for their anti-inflammatory activity against pro-inflammatory expression of iNOS and COX-2. The results demonstrated that compounds **3**–**5** exhibited inhibitory effects on LPS-induced iNOS in RAW264.7 cells in a dose-dependent manner (Figure 4C, D and E). However, all tested compounds were inactive against LPS-induced COX-2 in RAW264.7 cells.

## Conclusion

Six new and three known non-peptide small molecules were isolated from the millipede *Kronopolites svenhedini* (Verhoeff), and their structures were characterized using spectroscopic and ECD calculation methods. Biological evaluation of compounds **3**‒**5** showed inhibitory activities against LPS-induced iNOS in RAW264.7 cells. These findings contribute new insights into the chemistry and biological activity of arthropod-derived non-peptide small molecules.

## Experimental

### General

1D and 2D NMR spectra were acquired using Bruker AV-500 and AV-600 spectrometers (Bruker), with tetramethylsilane (TMS) used as an internal standard. HRESIMS data were obtained using a Shimazu LC-20 CE AB SCIEX QTOF X500R MS spectrometer (Shimadzu Corporation, Tokyo, Japan). Optical rotations (ORD) were collected with a Horiba SEPA-300 polarimeter. Ultraviolet (UV) and circular dichroism (CD) spectra were carried out on a Jasco J-815 CD spectrometer (JASCO). Column chromatography (CC) was performed using macroporous adsorbent resin Amberlite TM XAD 16N (particle size 20–60 mesh, Rohm and Haas Company), MCI gel CHP 20P (particle size 75–150 μm, Mitsubishi Chemical Industries, Japan), RP-18 (particle size 40–60 μm; Daiso Co.), C-18 silica gel (particle size 40–60 μm; Daiso Co., Japan), Sephadex LH-20 (Amersham Biosciences), and YMC gel ODS-A-HG (particle size 40–60 μm; YMC Co. Japan). A Saipuruisi chromatograph with a semi-preparative high-pressure infusion pump (SP-5030) and a semi-preparative UV–vis dual wavelength detector (UV200) were utilized for RP–HPLC. A YMC-Pack ODS-A column (250 mm × 20 mm, i.d., S-5 μm) was employed for preparative HPLC, while three columns (a YMC-Pack ODS-A column (250 mm × 10 mm, i.d., 5 μm), a Stabllity 100 C30 column (25 mm × 10 mm, i.d., 5 μm), and an Inetex-Biphenyl 100A column (250 mm × 10 mm, i.d., 5 μm)) were used for semi-preparative HPLC.

### Arthropod material

The dried arthropod bodies of *Kronopolites svenhedini* (Verhoeff) were purchased from Qunkang Pharmaceutical Co. in Anhui Province, PR China, in July 2021. The voucher specimen of this material (CHYX-0674) has been deposited at the School of Pharmaceutical Sciences, Health Science Center, Shenzhen University, PR China.

### Extraction and isolation

The dried and powdered bodies of *Kronopolites svenhedini* (Verhoeff) (49 kg) were extracted using 50% EtOH (4 × 120 L, 24 h each time) to yield a crude extract. This extract (5.2 kg) was partitioned into six fractions (Fr. A–Fr. F) utilizing a macroporous adsorbent resin column eluted with gradient aqueous MeOH (0–100%). Fr. E (180 g) was separated into ten parts (Fr. E1–Fr. E9, Fr. EA) using an MCI gel CHP 20P column (MeOH/H_2_O, 60–100%). Fr. E2 (2.0 g) was further divided into six portions (Fr. E21–Fr. E26) by Sephadex LH-20 (MeOH/H_2_O, 70%). Fr. E25 (248.6 mg) was subjected to preparative HPLC (MeOH/H_2_O (0.04% TFA), 50–100%, flow rate: 10 mL min^−1^) to obtain six fractions (Fr. E251–Fr. E256). Fr. E254 was concentrated under reduced pressure to yield compound **9** (20.00 mg). Additionally, Fr. E255 (51.8 mg) was further purified using semi-preparative HPLC (C30, MeOH/H_2_O, 25%, flow rate: 3 mL min^−1^) to yield compound **8** (16.23 mg, *t*_R_ = 25.04 min). Fr. E5 (9.0 g) was separated into eleven parts (Fr. E51–Fr. E59, Fr. E5A–B) by Sephadex LH-20 (MeOH/H_2_O, 70%). Fr. E53 (4.26 g) was divided into eleven portions (Fr. E531–Fr. E539, Fr. E53A, Fr. E53B) by using an MCI gel CHP 20P column (MeOH/H_2_O, 10–100%). Subsequently, Fr. E537 (354.8 mg) was divided into three parts (Fr. E5371–Fr. E5373) by Sephadex LH-20 (MeOH). Fr. E5373 (216.6 mg) was further fractionated into eight parts by a silica gel column (PE/EtOAc 2:1–1:1, to DCM/MeOH 20:1–1:1), and Fr. E53732 (63.8 mg) was further purified using semi-preparative HPLC (ODS-A, MeCN/H_2_O (0.04% TFA), 55%, flow rate: 3 mL min^−1^) to afford compound **7** (15.70 mg, *t*_R_ = 14.05 min). Fr. E54 (415.3 mg) was separated into twelve fractions (Fr. E541–Fr.E549, Fr. E54A–Fr.E54C) using an MCI gel CHP 20P column (MeOH/H_2_O, 30–100%), followed by semi-preparative HPLC (ODS-A, MECN/H_2_O (0.04% TFA), 28%, flow rate: 3 mL min^−1^) to obtain compound **1** (1.32 mg, *t*_R_ = 27.89 min). Fr. E8 (13.1 g) was partitioned into five portions (Fr. E81–Fr. E85) by Sephadex LH-20 (MeOH). Fr. E84 (207.3 mg) was divided into thirteen portions (Fr. E841–Fr. E849, Fr. E84A–Fr. E84D) using an ODS-A-HG column (MeOH/H_2_O, 30–100%). Fr. E846 (21.4 mg) was purified via semi-preparative HPLC (C30, MeCN/H_2_O (0.04% TFA), 42%, flow rate: 3 mL min^−1^) to afford compound **4** (0.97 mg, *t*_R_ = 51.45 min). Fr. E847 (21.4 mg) was purified through semi-preparative HPLC (ODS-A, MeCN/H_2_O (0.04% TFA), 40%, flow rate: 3 mL min^−1^) to afford compound **5** (1.21 mg, *t*_R_ = 56.80 min) and compound **6** (1.16 mg, *t*_R_ = 61.32 min). Fr. E85 (686.7 mg) was separated into ten fractions (Fr. E851–Fr. E859, Fr. E85A) using an RP-18 column (MeOH/H_2_O, 30–100%). Fr. E856 (31.3 mg) was purified using semi-preparative HPLC (Inetex-Biphenyl, MECN/H_2_O (0.04% TFA), 38%, flow rate: 3 mL min^−1^) to afford compounds **3** (0.99 mg, *t*_R_ = 25.33 min) and **2** (0.76 mg, *t*_R_ = 30.38 min).

### Compound characterization

**Kronopoone A** (**1**): yellow gum; [α]_D_^25^ +9.38 (*c* 0.32, MeOH); UV (MeOH) λ_max_, nm (log ε): 208 (2.92), 229 (2.88), 275 (2.78); ECD (MeOH) λ, nm (Δε): 210 (−1.88), 231 (+0.34), 251 (+0.02), 273 (−0.41), 327 (+0.39); HRESIMS (*m*/*z*): [M + H]^+^ calcd for C_14_H_19_O_4_, 251.1278; found, 251.1274; ^1^H and ^13^C NMR data, see Table 1.

**Kronopoiol A** (**2**): brown solid; UV (MeOH) λ_max_, nm (log ε): 202 (3.56), 242 (3.58), 280 (3.13), 324 (3.15), 367 (2.89); HRESIMS (*m*/*z*): [M + H]^+^ calcd for C_17_H_17_O_6_, 317.1020; found 317.1008 ; ^1^H and ^13^C NMR data, see Table 2.

**Kronopoiol B** (**3**): brown solid; UV (MeOH) λ_max_, nm (log ε): 201 (3.18), 241 (3.44), 260 (3.22), 324 (2.73), 378 (2.69); HRESIMS (*m*/*z*): [M + H]^+^ calcd for C_15_H_13_O_4_, 257.0808; found, 257.0802 ; ^1^H and ^13^C NMR data, see Table 2.

**5-*O*-Methyldaphnegiralin C_1_** (**4**): brown solid; [α]_D_^25^ +30.77 (*c* 0.26, MeOH); UV (MeOH) λ_max_, nm (log ε): 207 (3.92), 280 (3.14); ECD (MeOH) λ, nm (Δε): 208 (+15.19), 237 (−1.90), 250 (+0.23), 283 (−0.71); HRESIMS (*m*/*z*): [M + H]^+^ calcd for C_21_H_25_O_5_, 357.1697; found, 357.1680; ^1^H and ^13^C NMR data, see Table 3.

**Kronoponoid A** (**7**): light yellow gum; [α]_D_^25^ +7.50 (*c* 0.40, MeOH); UV (MeOH) λ_max_, nm (log ε): 202 (3.68); HRESIMS (*m*/*z*): [M + H]^+^ calcd for C_16_H_29_O_3_, 269.2111; found, 269.2116 ; ^1^H and ^13^C NMR data, see Table 4.

**Kronoponoid B** (**8**): light yellow gum; [α]_D_^25^ +2.50 (*c* 0.40, MeOH); UV (MeOH) λ_max_, nm (log ε): 202 (3.81); HRESIMS (*m*/*z*): [M + H]^+^ calcd for C_17_H_31_O_3_, 283.2268; found, 283.2268 ; ^1^H and ^13^C NMR data, see Table 4.

### Computational methods

The CONFLEX 7 searches, considering the Molecular Merck force field (MMFF94) and DFT/TDDFT calculations, were performed for model compounds (3*R,*4*R)*-**1**, (3S*,*4*S)*-**1**, (2*S,*2*″R)*-**4**, (2*S,*2*″S)*-**4**, (2*R,*2*″S)*-**4**, and (2*R,*2*″R)*-**4** using the Spartan'14 software package and the Gaussian 09 program package. The ECD calculations of the predominant conformers (80%) were conducted using DFT calculations at the B3LYP/6-311G(d,p) level of theory. The program SpecDis 1.62 was used to generate the CD spectra [31].

### Biological evaluation

#### Antitumor assay

Panc02-h7-GP-GFP cells (derived from the transformation of mouse pancreatic cancer cell line Panc02-h7) were maintained at 37 °C in a 5% CO_2_ atmosphere using high-glucose DMEM (GIBCO, USA) containing 10% fetal bovine serum (FBS, GIBCO, USA), 100 U/mL penicillin, 10 μg/mL streptomycin, and 10 μg/mL puromycin. Cells were seeded at a density of 5000 cells/well in 96-well plates under the same incubation conditions. Following overnight culture, cells were pretreated with compounds at a 20 μM concentration or DMSO for 48 h, with gemcitabine as a positive reference drug. Subsequently, Cell Count Kit-8 (CCK-8, MCE, USA) was added to each well at a 10 μL concentration for 2 h. The absorbance at 450 nm was measured using a microplate reader (TECAN, Switzerland).

#### Antitumor activity assay of CD8^+^ T cells in vitro

This assay, along with the associated experimental procedures, received approval from the Institutional Animal Care and Use Committee of Shenzhen University Health Science Center and the Animal Experimentation Ethics Committee of Shenzhen University Health Science Center (AEWC-202300026). All animal housing and handling adhered to the research ethics guidelines set forth by the Institutional Animal Care and Use Committee of Shenzhen University Health Science Center and the Animal Experimentation Ethics Committee of Shenzhen University Health Science Center.

Panc02-h7-GP-GFP cells were treated with trypsin (0.25%, Sigma) and resuspended in PBS (GIBCO, USA) following centrifugation. The cell suspension was then injected into the pancreas of mice (1 × 10^6^ cells per mouse). After 14 days, tumors were excised, digested with a digestion solution, ground, and centrifuged to produce a cell suspension. Lymphocytes were isolated from the cell suspension using the Percoll method and were further enriched (via negative selection) to obtain naive CD8^+^ T cells. The enrichment effect and phenotype of the CD8^+^ cells were detected by flow cytometry (BD, USA). CD8^+^ T cells and Panc02-h7-GP-GFP cells were co-cultured with the corresponding concentrations of compounds or DMSO for 18 h. Fluorescence intensity was measured using a microplate reader (emission at 476 nm, excitation at 514 nm).

#### Anti-inflammatory assay

RAW264.7 (a mouse macrophage cell line) cells were incubated in high-glucose DMEM (GIBCO, USA) containing 10% fetal bovine serum (FBS, GIBCO, USA), 100 U/mL penicillin, and 100 μg/mL streptomycin. Cells were maintained in an atmosphere of 5% CO_2_ at 37 °C and subsequently plated in 96-well plates at a concentration of 2 × 10^4^ cells/mL under the same incubation conditions. Following overnight incubation, the cells were treated with the corresponding concentration of the compound or DMSO for 24 h. CCK-8 (Beyotime, China) solution was added and incubated for 1 h. The absorbance of the solution in the 96-well plate was measured using a microplate reader (450 nm, BioTek, USA), and the cell survival rate was calculated.

Western blot analysis was used to detect protein levels in cells. RAW264.7 cells were pre-treated with the corresponding concentration of the compound or DMSO for 2 h and then stimulated with lipopolysaccharide (LPS, 1 μg/mL) for 12 h. Subsequently, radioimmunoprecipitation assay (RIPA) buffer (Beyotime, PR China) containing a protease inhibitor (Roche, Germany) was used to extract total protein from cells. Protein content samples was determined by the BCA assay (Thermo, USA).

Equivalent protein extracts were separated by 10% SDS-PAGE and transferred onto PVDF membranes. The membranes were blocked with 5% BSA and incubated with the indicated antibodies overnight at 4 °C. Following this, the membranes were incubated with horseradish peroxidase (HRP)-conjugated secondary antibody at room temperature. The ECL kit (Pierce, USA) and analysis system (Bio-Rad, CA, USA) were used to visualize and detect the bands. Immunoblot densitometric analysis results were processed using ImageJ software (NIH, USA).

## Supporting Information

File 1 NMR, HRESIMS, and CD spectra for new compounds and the figures of antitumor activity assay of CD8^+^ T cells in vitro.
