# Correction: Non-peptide compounds from *Kronopolites svenhedini* (Verhoeff) and their antitumor and iNOS inhibitory activities

**DOI:** 10.3762/bjoc.19.97

**Published:** 2023-09-11

**Authors:** Yuan-Nan Yuan, Jin-Qiang Li, Hong-Bin Fang, Shao-Jun Xing, Yong-Ming Yan, Yong-Xian Cheng

**Affiliations:** 1 School of Pharmacy, Guangdong Pharmaceutical University, Guangzhou 510006, PR China; 2 Institute for Inheritance-Based Innovation of Chinese Medicine, School of Pharmaceutical Sciences, Health Science Center, Shenzhen University, Shenzhen 518060, PR China; 3 Department of Pathogen Biology, Health Science Center, Shenzhen University, Shenzhen 518060, PR China

**Keywords:** arthropod, iNOS, *Kronopolites svenhedini* (Verhoeff), non-peptide small molecules

The structure of compound **1** of the original publication was misattributed and should be revised as shown in Figure 1. The error happened due to insufficient in-depth 2D NMR analysis. We reanalyzed the 2D NMR data of compound **1** in detail and finally determined the correct structure as shown in Figure 1. The revised structure of **1** is supported by the HMBC correlations of H-2/C-1, C-3, C-4, C-8a, H-4/C-3, C-4a, C-5, C-8a, C-9, H-5/C-4, C-4a, C-6, C-7, C-8a, H-9/C-2, C-3, C-4, H-10/C-7, C-8, C-8a, H-11/C-6, and H-12/C-7.

**Figure 1:**
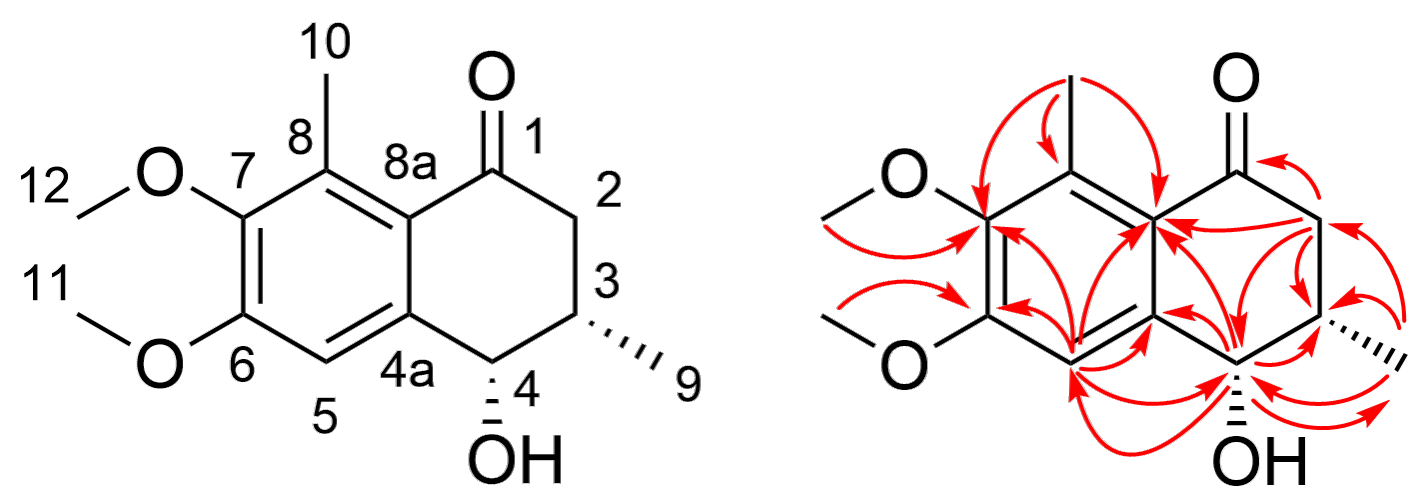
Revised structure of compound **1** and key HMBC correlations.

Table 1 provides the revised 1D ^1^H and ^13^C NMR data of compound **1**.

**Table 1:** Revised ^1^H (600 MHz) and ^13^C NMR (150 MHz) data of compound **1** (δ in ppm, *J* in Hz, methanol-*d*_4_).

No.	δ_H_ (mult, *J*, amount)	δ_C_ mult	No.	δ_H_ (mult, *J*, amount)	δ_C_ mult

C-1		201.3 C	C-7		148.5 C
C-2	2.64 (dd, *J* = 17.2, 10.3, 1H) 2.48 (dd, *J* = 17.2, 4.7, 1H)	43.7 CH_2_	C-8		136.1 C
C-3	2.36 (m, 1H)	35.8 CH	C-8a		124.5 C
C-4	4.69 (d, *J* = 3.0, 1H)	72.9 CH	C-9	1.09 (d, *J* = 6.8, 3H)	16.3 CH_3_
C-4a		145.6 C	C-10	2.51 (s, 3H)	14.1 CH_3_
C-5	7.02 (s, 1H)	110.8 CH	C-11	3.96 (s, 3H)	56.3 CH_3_
C-6		158.0 C	C-12	3.73 (s, 3H)	60.7 CH_3_

The structural revision of **1** also required recalculation of the theoretical ECD spectra of both enantiomers to determine the absolute configuration. By comparison of the recalculated and the experimental spectra, it became evident that in fact, the (3*S*,4*S*)-enantiomer rather than the (3*R*,4*R*)-enantiomer was obtained (Figure 2).

**Figure 2:**
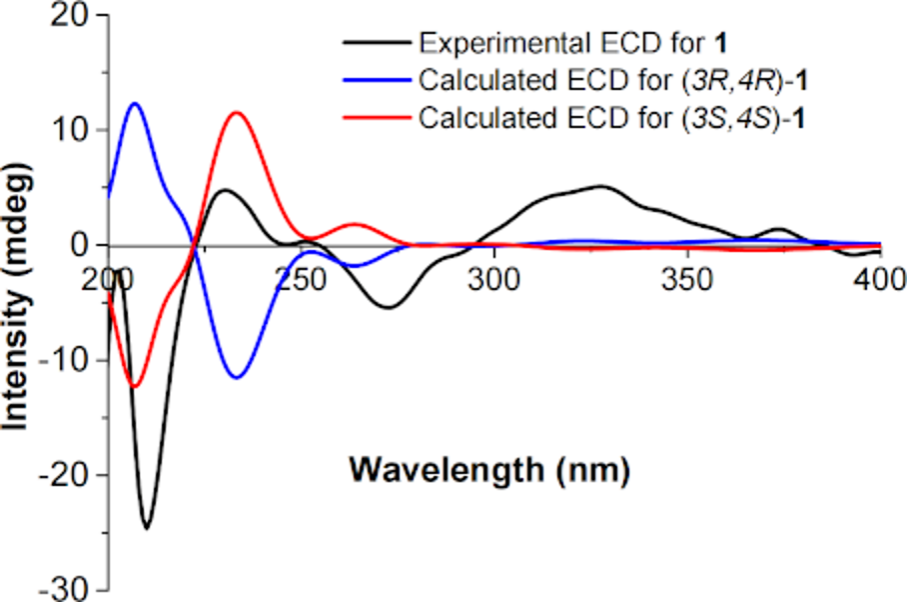
Recalculated and experimental ECD spectra of compound **1**.

Consequently, in the first paragraph of the Results and Discussion section in the original publication, the sentence “The HMBC correlations […] disclosed that C-10 is connected to C-5 in compound **1**.” is inaccurate. Following the reanalysis of the HMBC correlations, it is now evident that C-10 is connected to C-8 in compound **1**.

