# Supplementary material for: Non-peptide compounds from Kronopolites svenhedini (Verhoeff) and their antitumor and iNOS inhibitory activities
Source: Beilstein J Org Chem. 2023 Jun 7;19:789–99. doi: 10.3762/bjoc.19.59 (PMC10280063; doi:10.3762/bjoc.19.59)
Supplement: File 1 — NMR, HRESIMS, and CD spectra for new compounds and the figures of antitumor activity assay of CD8+ T cells in vitro. [file Beilstein_J_Org_Chem-19-789-s001.pdf]

BEILSTEIN JOURNAL OF ORGANIC CHEMISTRY

Supporting Information

for

Non-peptide compounds from *Kronopolites svenhedini* (Verhoeff) and their antitumor and iNOS inhibitory activities

Yuan-Nan Yuan, Jin-Qiang Li, Hong-Bin Fang, Shao-Jun Xing, Yong-Ming Yan and Yong-Xian Cheng

Beilstein J. Org. Chem. **2023,** *19,* 789–799. doi:10.3762/bjoc.19.59

NMR, HRESIMS, and CD spectra for new compounds and the figures of antitumor activity assay of $CD8^+$ T cells in vitro

License and Terms: This is a supporting information file under the terms of the Creative Commons Attribution License (https://creativecommons.org/licenses/by/4.0). Please note that the reuse, redistribution and reproduction in particular requires that the author(s) and source are credited and that individual graphics may be subject to special legal provisions.
The license is subject to the *Beilstein Journal of Organic Chemistry* terms and conditions: (https://www.beilstein-journals.org/bjoc/terms)

Table of contents

Figure S1. ^{1}H NMR spectrum of **1** in methanol-d_4
Figure S2. ^{13}C NMR and DEPT spectrum of **1** in methanol-d_4
Figure S3. ^{1}H–^{1}H COSY spectrum of **1** in methanol-d_4
Figure S4. HSQC spectrum of **1** in methanol-d_4
Figure S5. HMBC spectrum of **1** in methanol-d_4
Figure S6. HRESIMS spectrum of **1**
Figure S7. CD spectrum of **1** in MeOH
Figure S8. ^{1}H NMR spectrum of **2** in methanol-d_4
Figure S9. ^{13}C NMR and DEPT spectrum of **2** in methanol-d_4
Figure S10. ^{1}H–^{1}H COSY spectrum of **2** in methanol-d_4
Figure S10. HSQC spectrum of **2** in methanol-d_4
Figure S12. HMBC spectrum of **2** in methanol-d_4
Figure S13. HRESIMS spectrum of **2**
Figure S14. ^{1}H NMR spectrum of **3** in methanol-d_4
Figure S15. ^{13}C NMR and DEPT spectrum of **3** in methanol-d_4
Figure S16. ^{1}H–^{1}H COSY spectrum of **3** in methanol-d_4
Figure S17. HSQC spectrum of **3** in methanol-d_4
Figure S18. HMBC spectrum of **3** in methanol-d_4
Figure S19. HRESIMS spectrum of **3**
Figure S20. ^{1}H NMR spectrum of **4** in methanol-d_4
Figure S21. ^{13}C NMR and DEPT spectrum of **4** in methanol-d_4
Figure S22. ^{1}H–^{1}H COSY spectrum of **4** in methanol-d_4
Figure S23. HSQC spectrum of **4** in methanol-d_4
Figure S24. HMBC spectrum of **4** in methanol-d_4
Figure S25. HRESIMS spectrum of **4**
Figure S26. CD spectrum of **4** in MeOH
Figure S27. ^{1}H NMR spectrum of **7** in methanol-d_4
Figure S28. ^{13}C NMR and DEPT spectrum of **7** in methanol-d_4
Figure S29. ^{1}H–^{1}H COSY spectrum of **7** in methanol-d_4
Figure S30. HSQC spectrum of **7** in methanol-d_4
Figure S31. HMBC spectrum of **7** in methanol-d_4
Figure S32. ROESY spectrum of **7** in methanol-d_4
Figure S33. HRESIMS spectrum of **7**
Figure S34. ^{1}H NMR spectrum of **8** in methanol-d_4
Figure S35. ^{13}C NMR and DEPT spectrum of **8** in methanol-d_4
Figure S36. ^{1}H–^{1}H COSY spectrum of **8** in methanol-d_4
Figure S37. HSQC spectrum of **8** in methanol-d_4
Figure S38. HMBC spectrum of **8** in methanol-d_4
Figure S39. ROESY spectrum of **8** in methanol-d_4
Figure S40. HRESIMS spectrum of **8**
Figure S41. Compounds inhibited cell proliferation of Panc02-h7-GP-GFP cells.
Figure S42. Purification of CD8+T cells in vitro.
Figure S43. Compounds affected the antitumor activity of $CD8^+$ T cells in vitro.

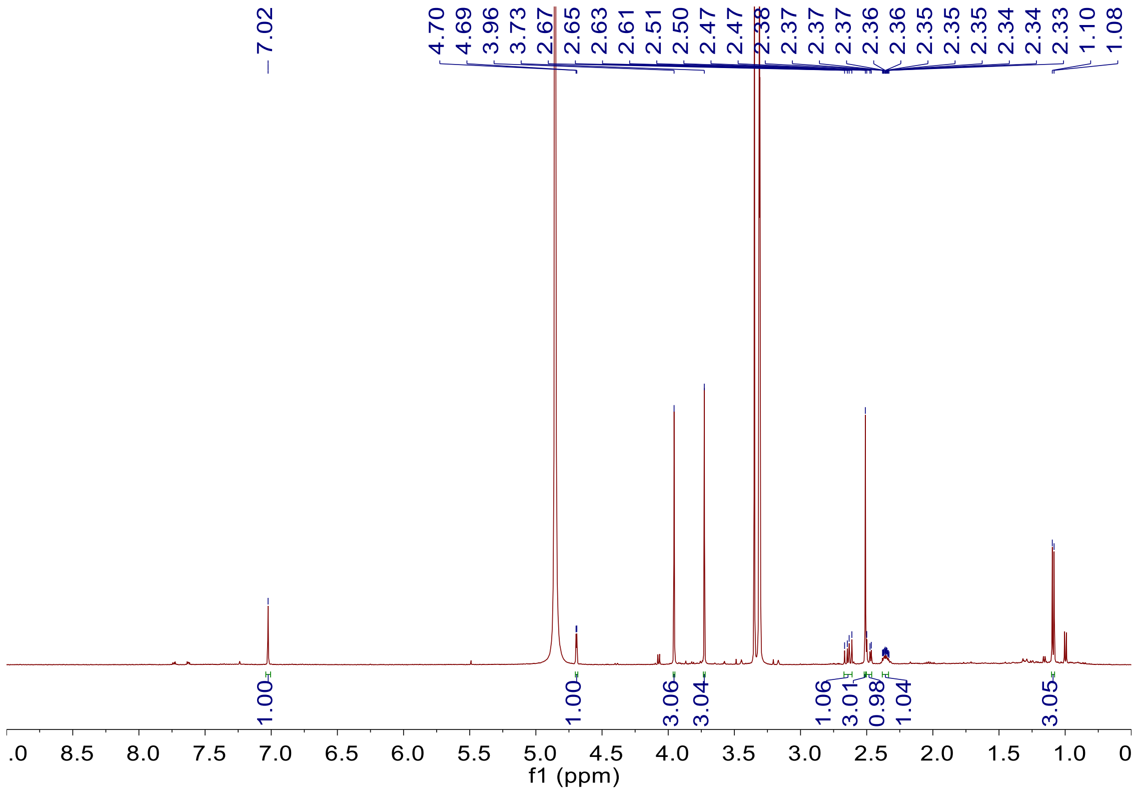

Figure S1: ^{1}H NMR spectrum of **1** in methanol-d_4

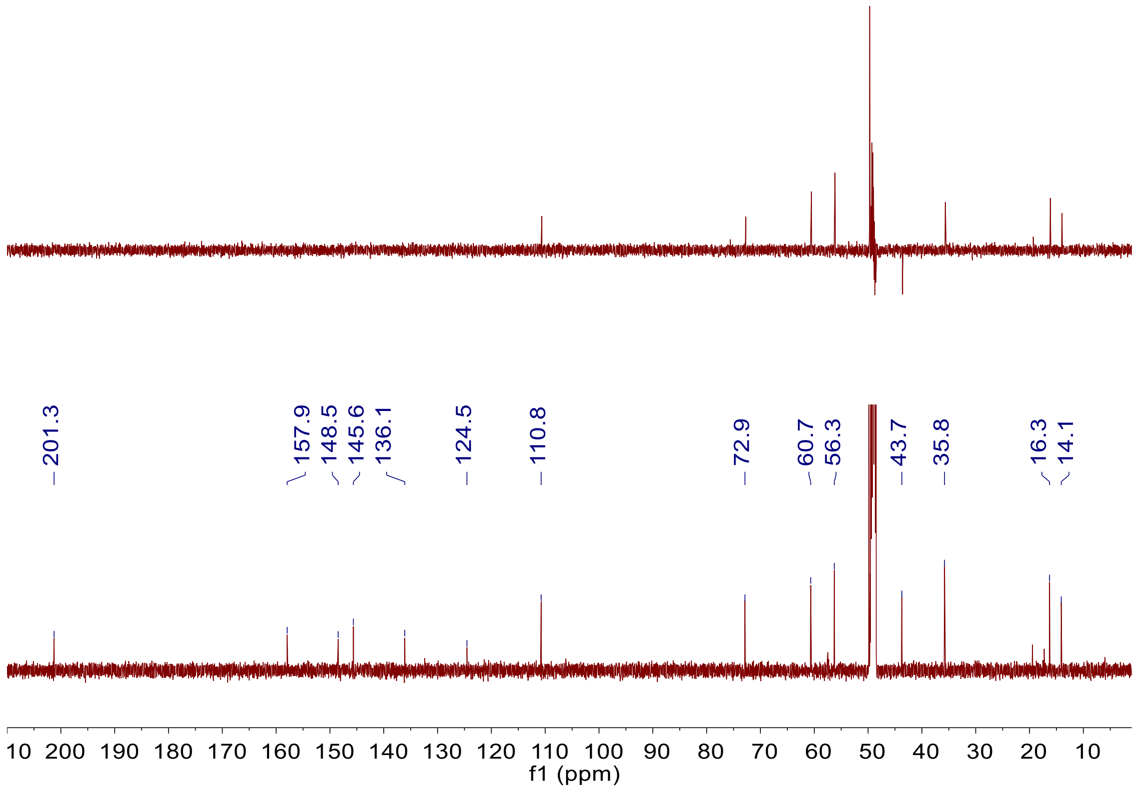

Figure S2: ^{13}C NMR and DEPT spectrum of **1** in methanol-d_4

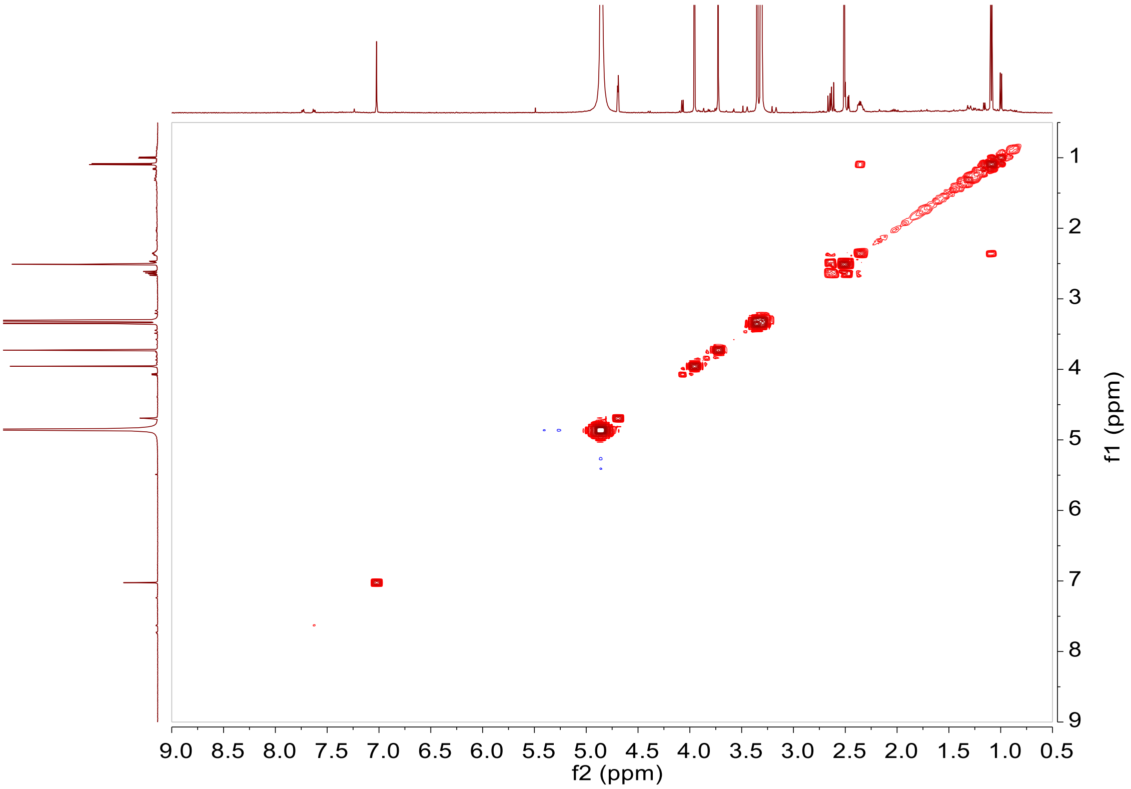

Figure S3: ^{1}H–^{1}H COSY spectrum of **1** in methanol-d_4

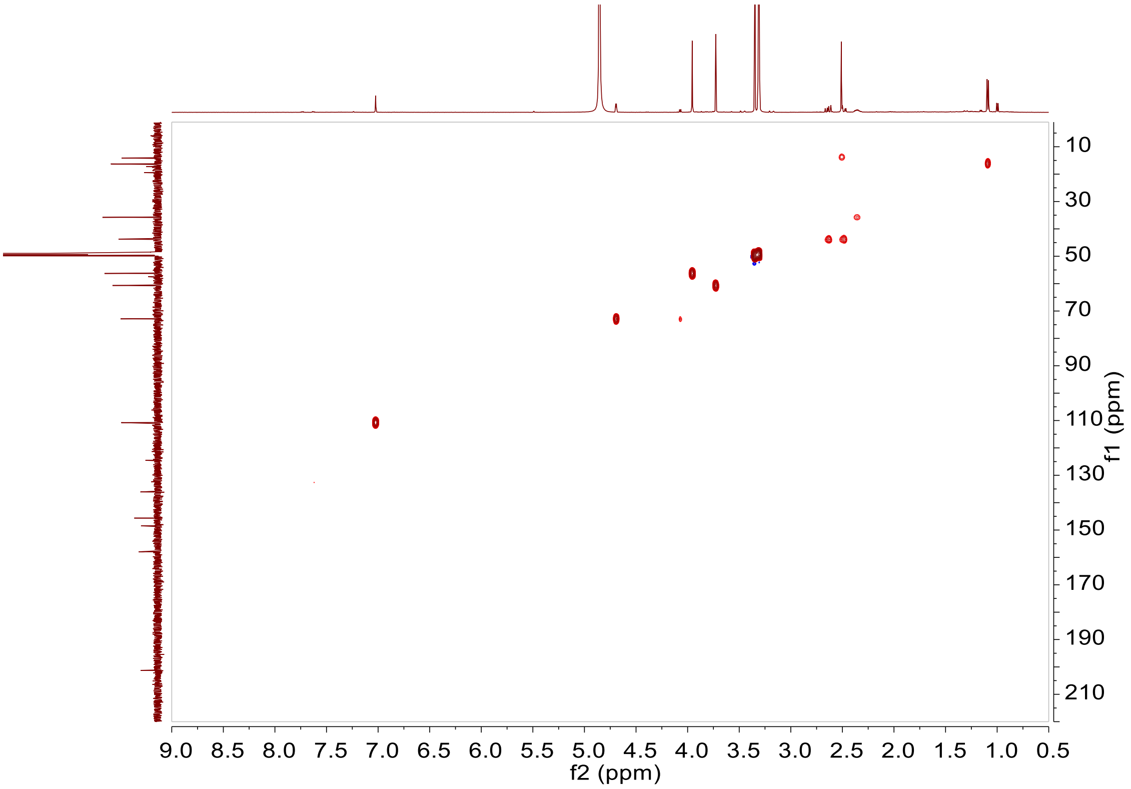

Figure S4: HSQC spectrum of **1** in methanol-d_4

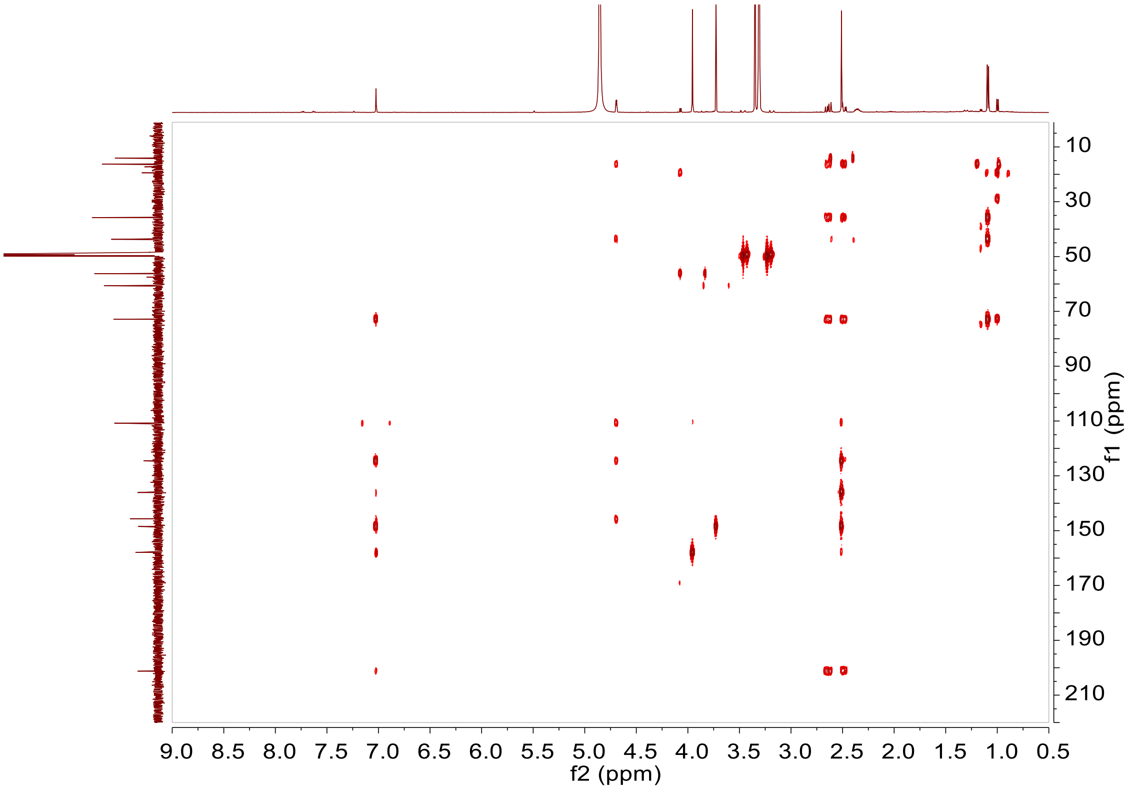

Figure S5: HMBC spectrum of **1** in methanol-d_4

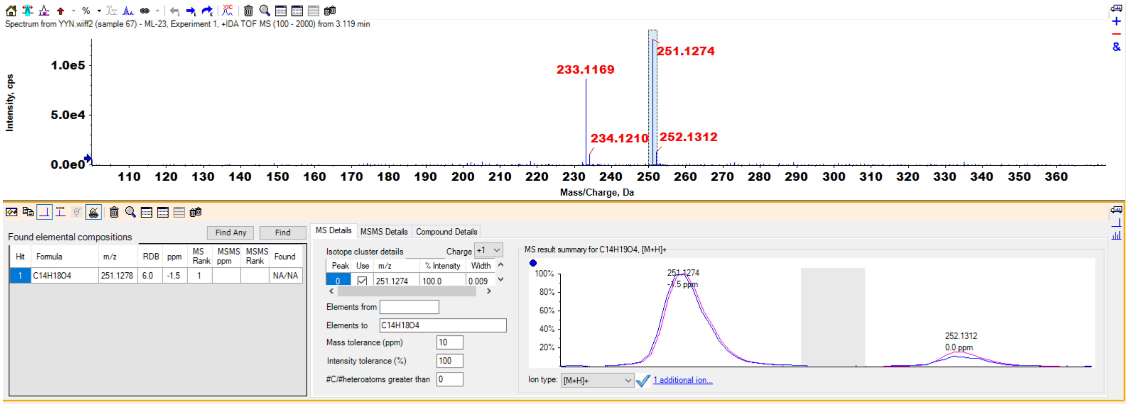

Figure S6: HRESIMS spectrum of **1**

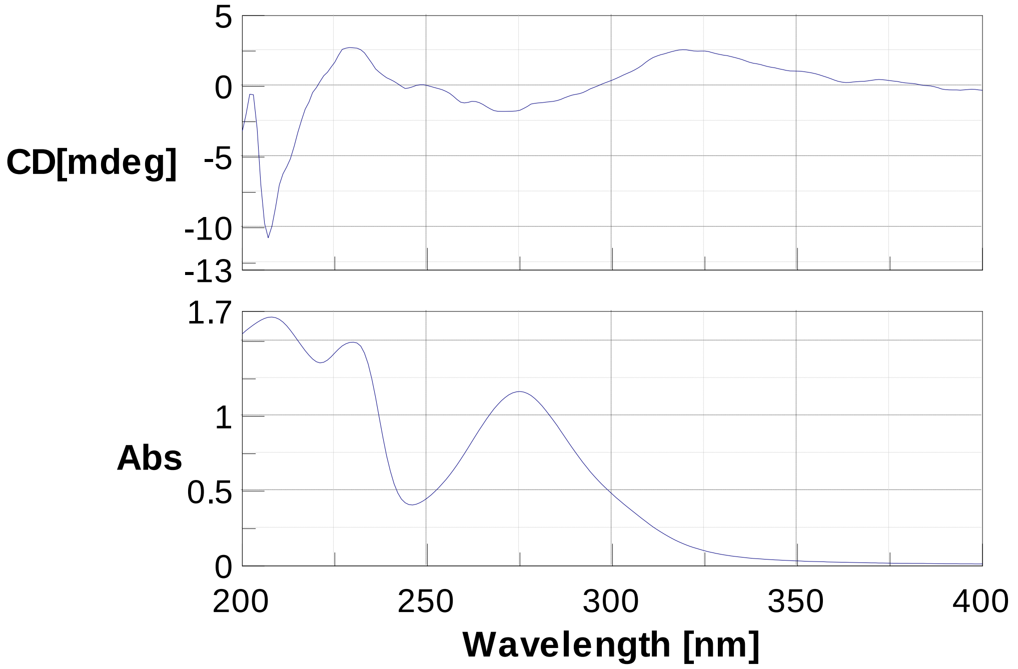

Figure S7: CD spectrum of **1** in MeOH

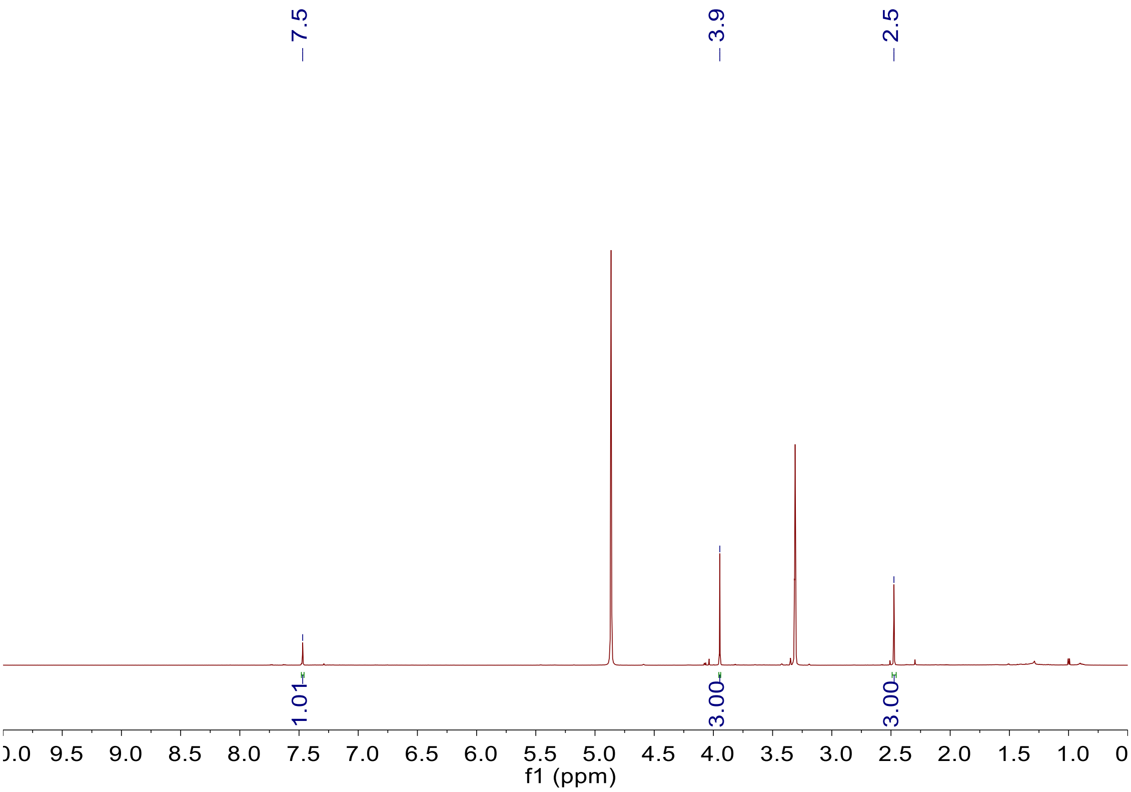

Figure S8: ^{1}H NMR spectrum of **2** in methanol-d_4

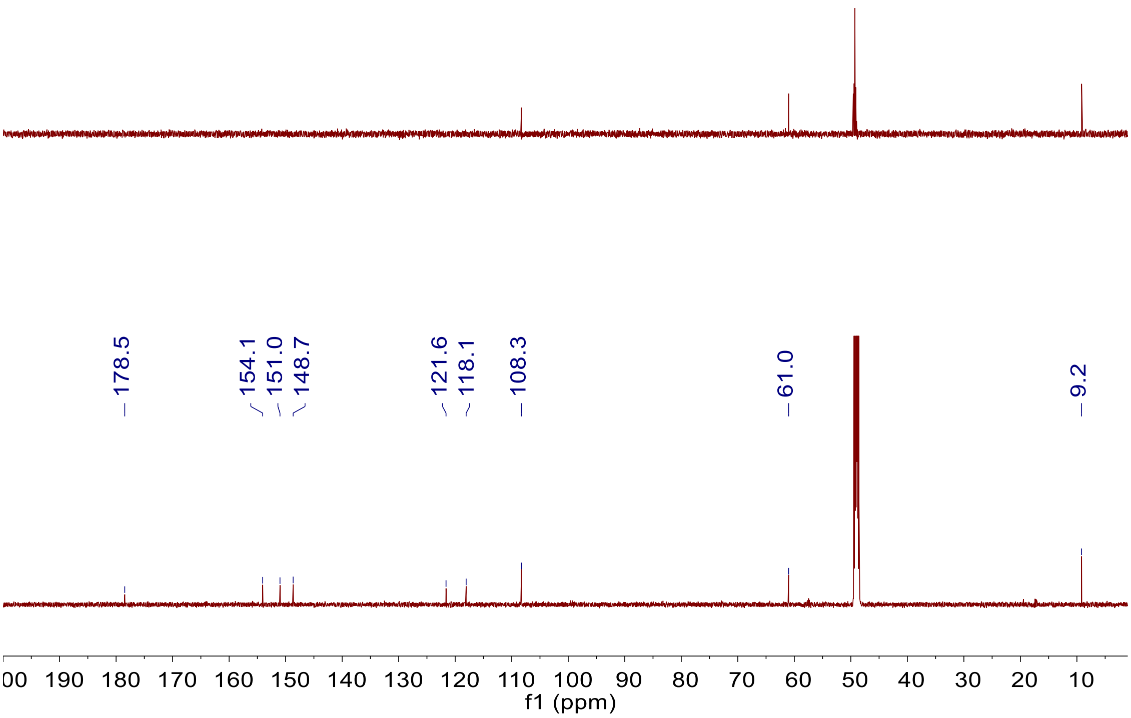

Figure S9: ^{13}C NMR and DEPT spectrum of **2** in methanol-d_4

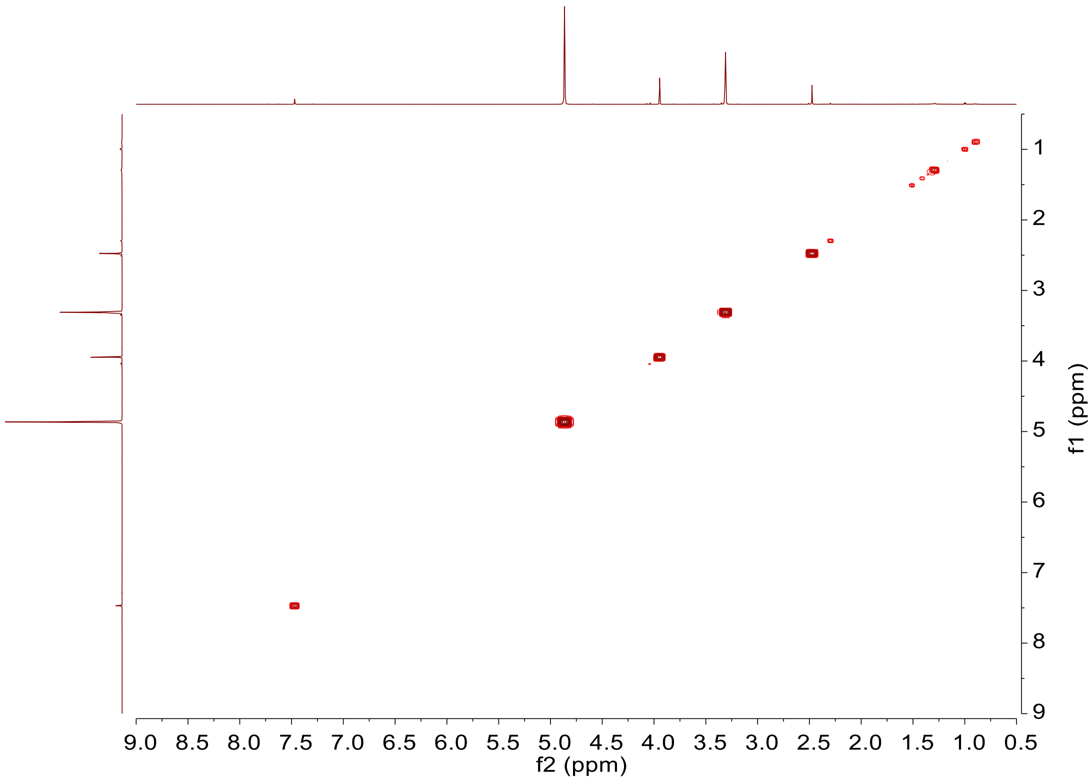

Figure S10: ^{1}H–^{1}H COSY spectrum of **2** in methanol-d_4

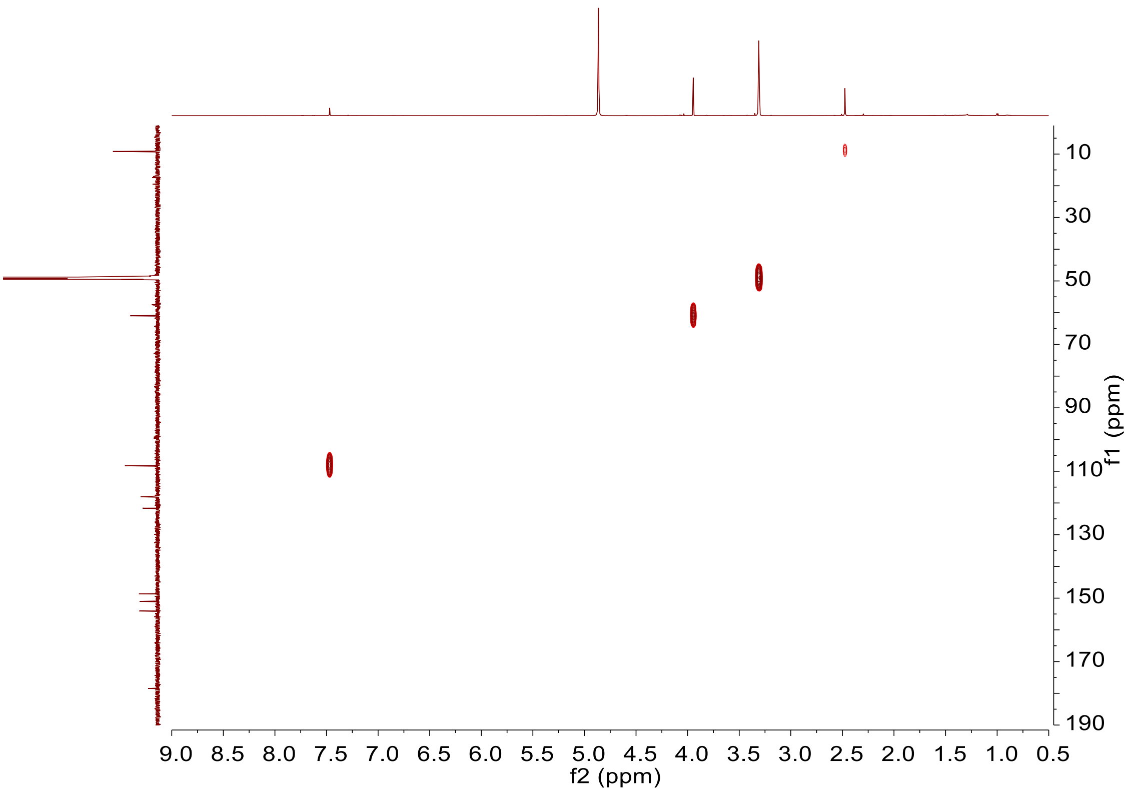

Figure S11: HSQC spectrum of **2** in methanol-d_4

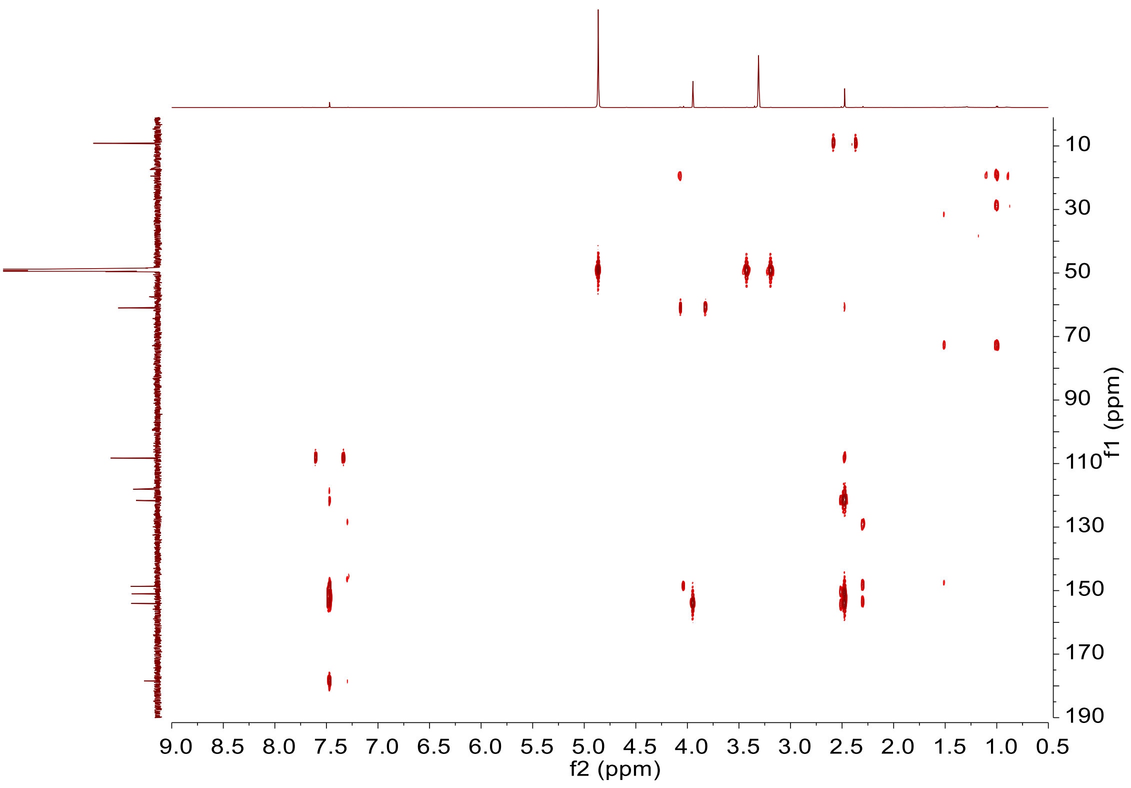

Figure S12: HMBC spectrum of **2** in methanol-d_4

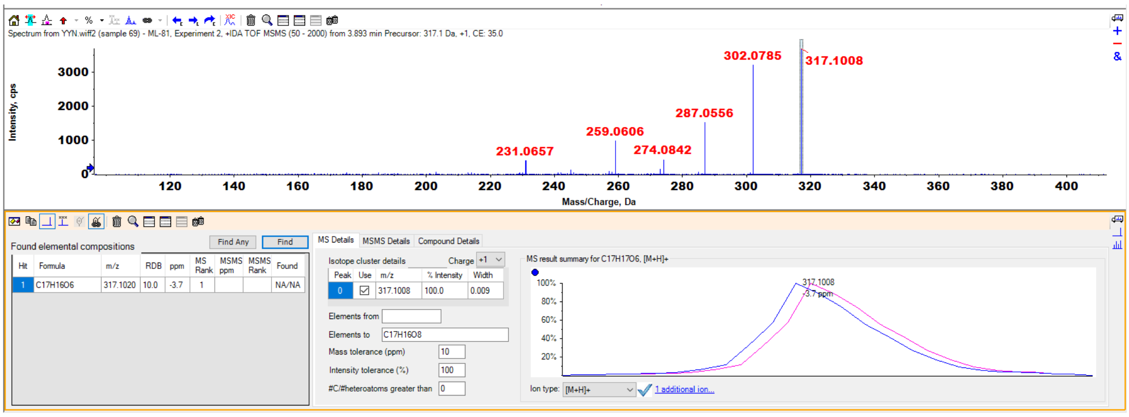

Figure S13: HRESIMS spectrum of **2**

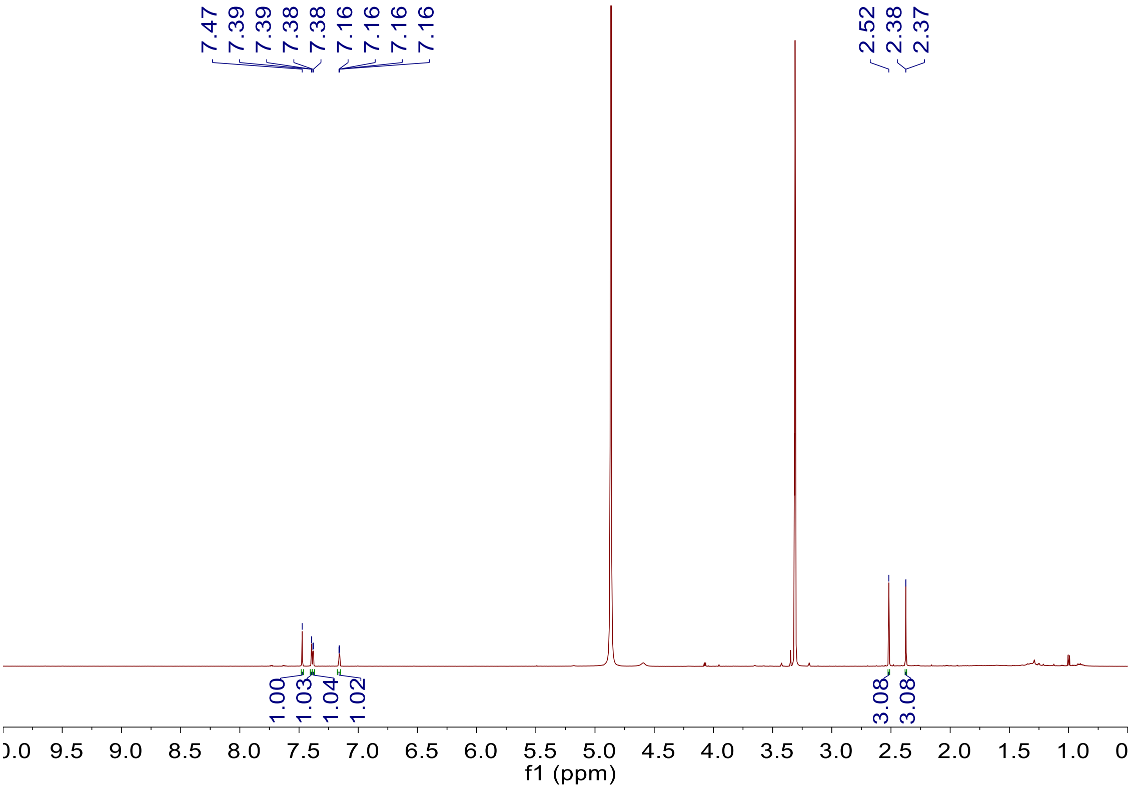

Figure S14: ^{1}H NMR spectrum of **3** in methanol-d_4

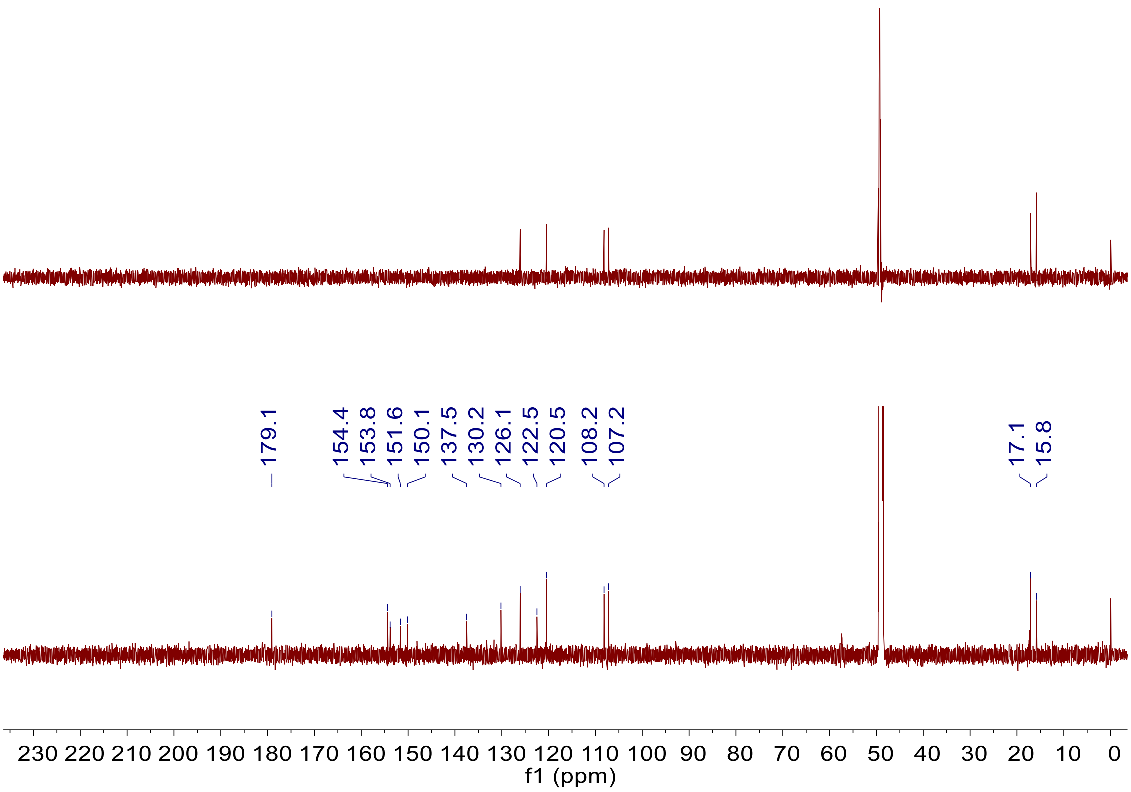

Figure S15: ^{13}C NMR and DEPT spectrum of **3** in methanol-d_4

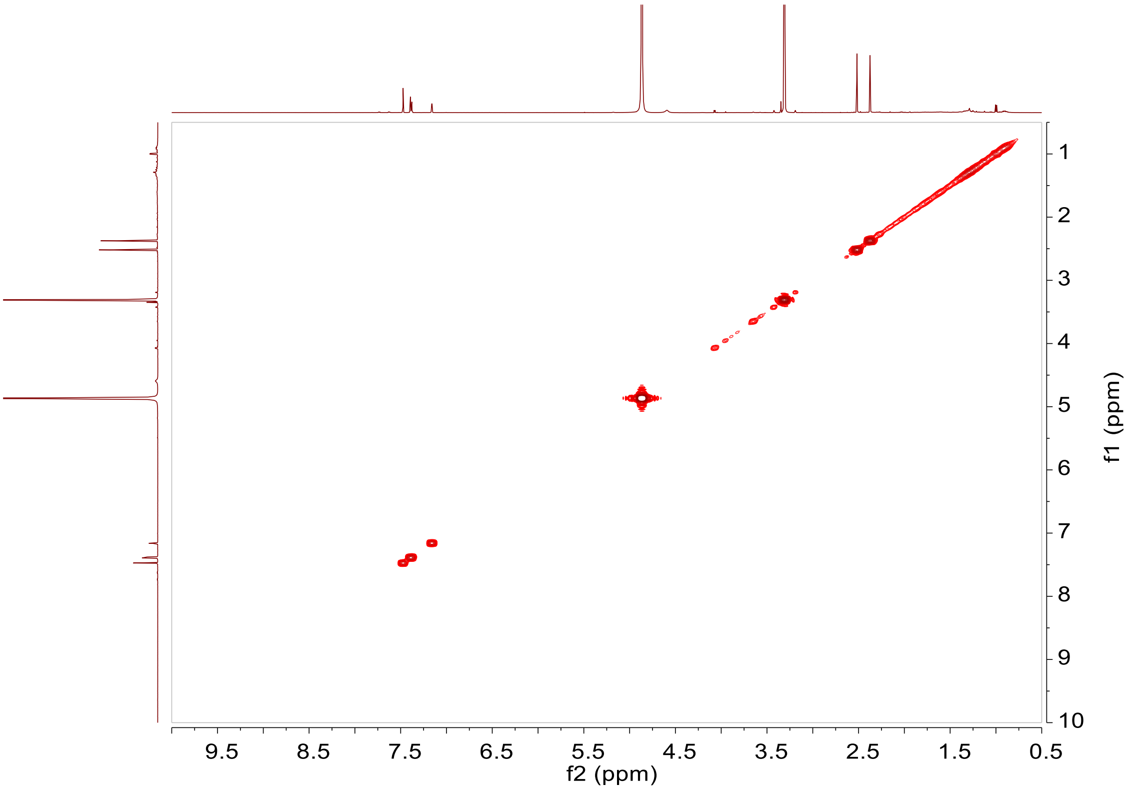

Figure S16: ^{1}H–^{1}H COSY spectrum of **3** in methanol-d_4

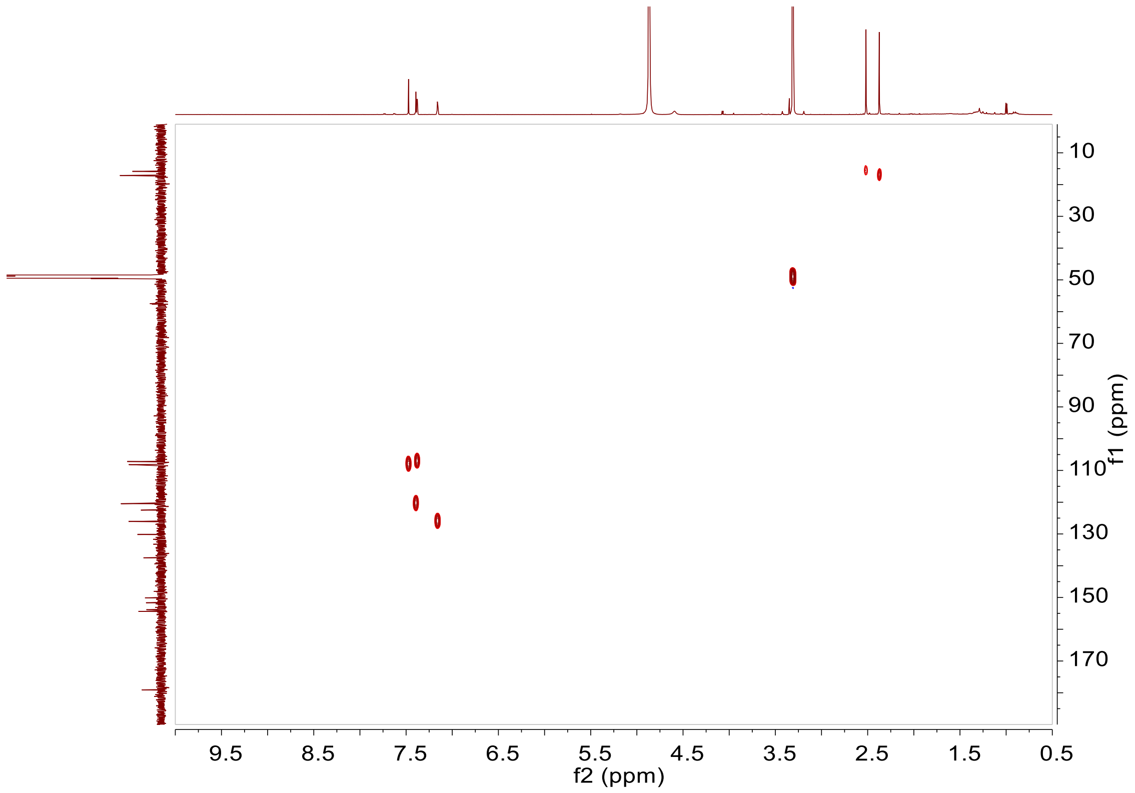

Figure S17: HSQC spectrum of **3** in methanol-*d*4

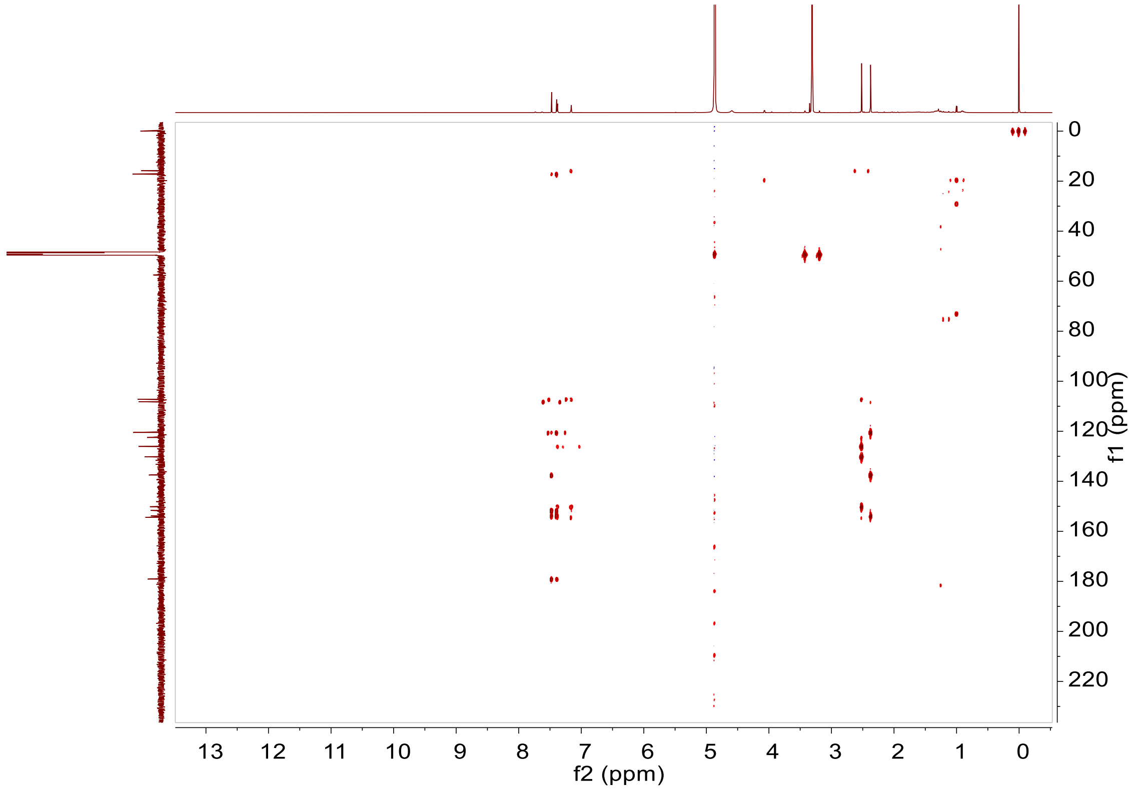

Figure S18: HMBC spectrum of **3** in methanol-*d*4

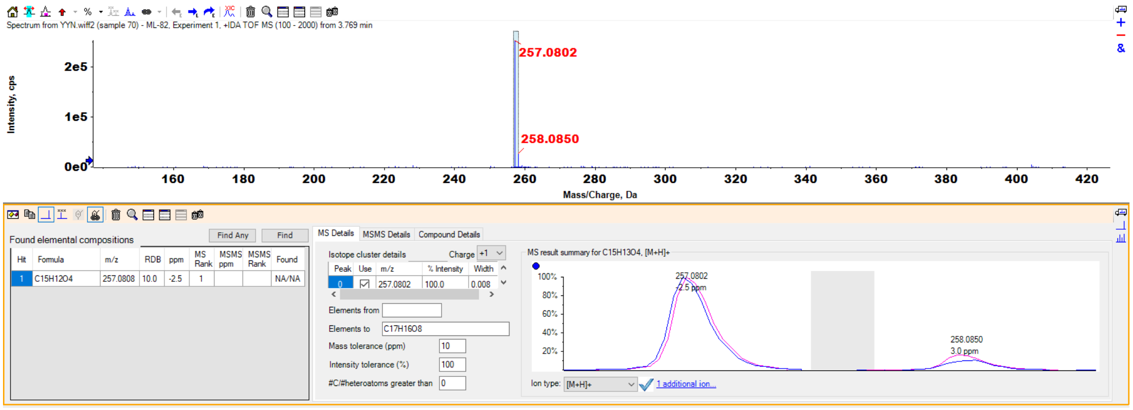

Figure S19: HRESIMS spectrum of **3**

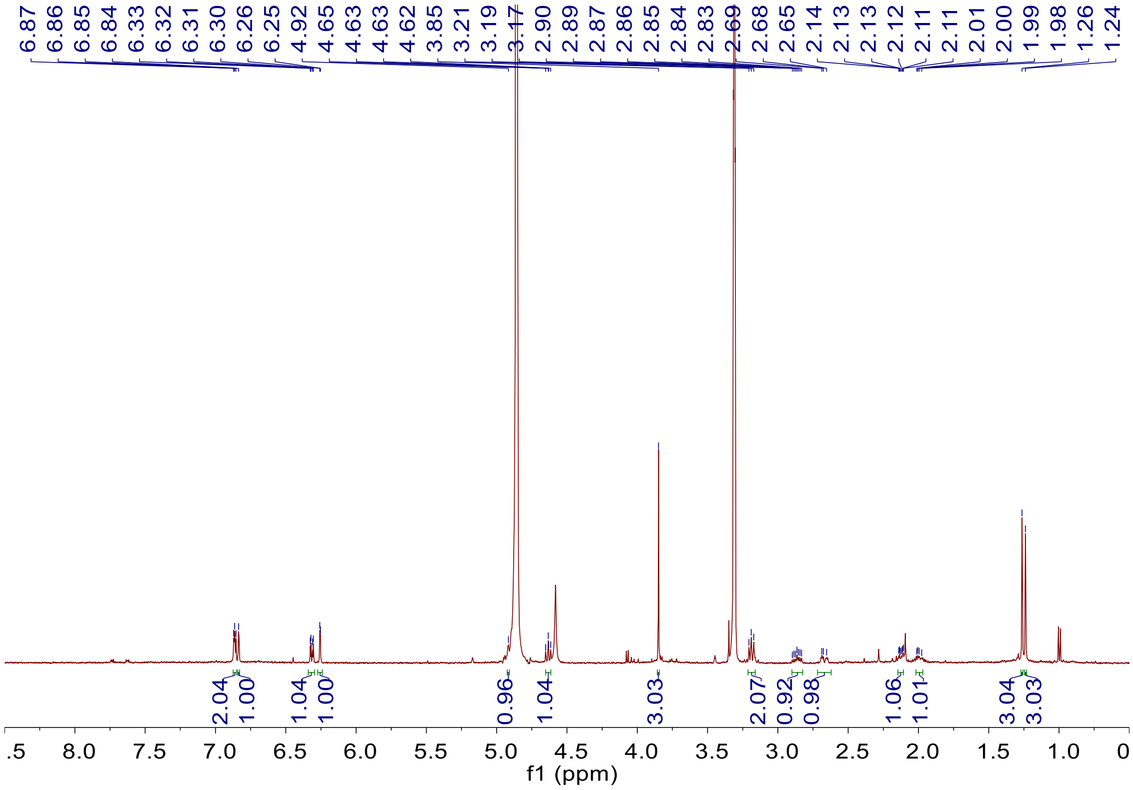

Figure S20: ^{1}H NMR spectrum of **4** in methanol-d_4

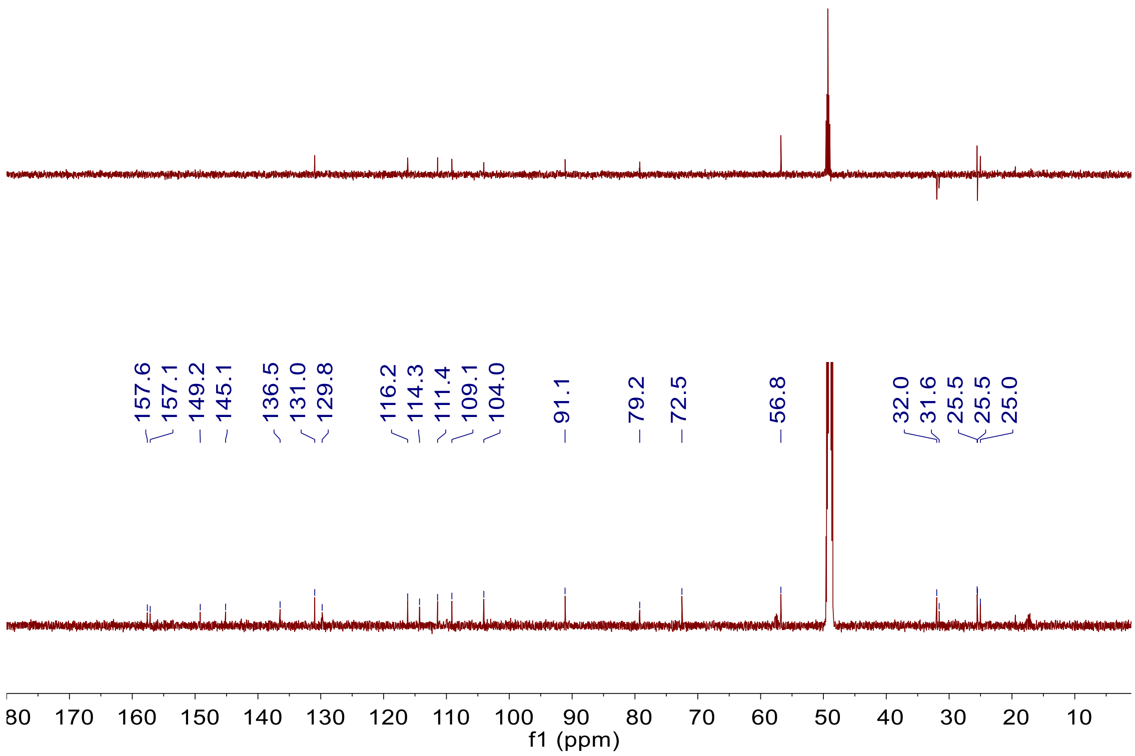

Figure S21: ^{13}C NMR and DEPT spectrum of **4** in methanol-*d*4

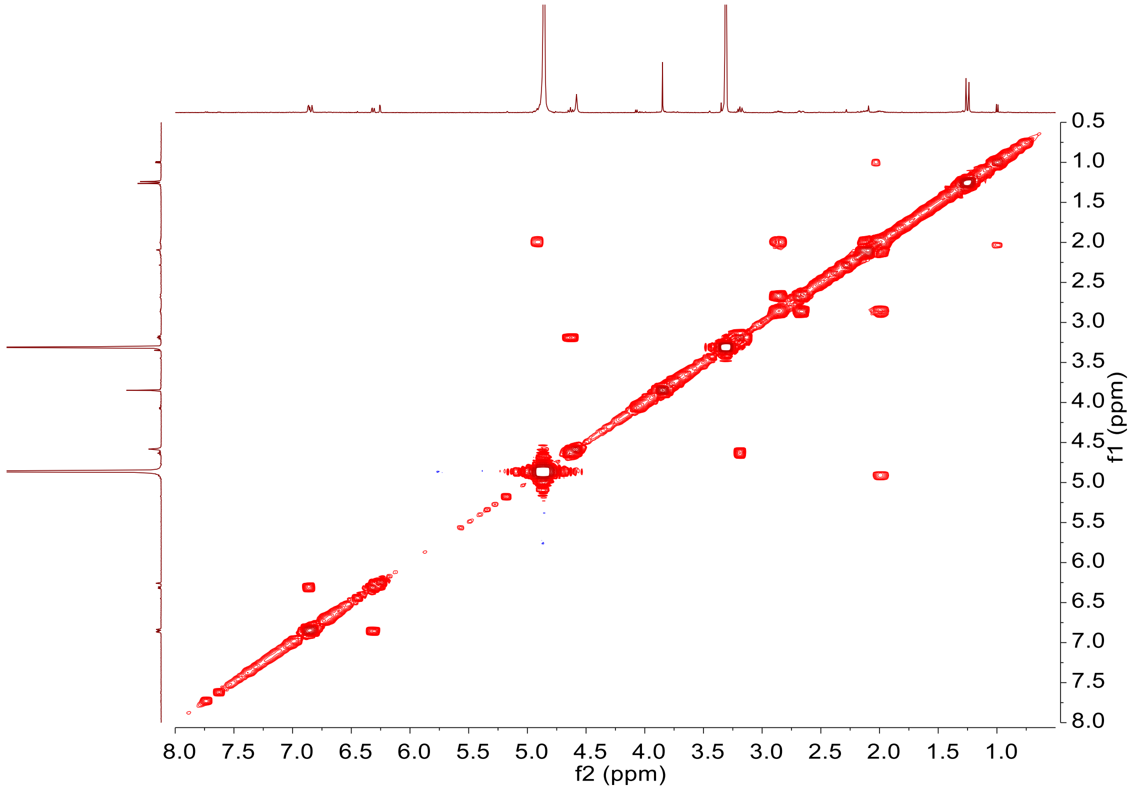

Figure S22: ^{1}H–^{1}H COSY spectrum of **4** in methanol-*d*4

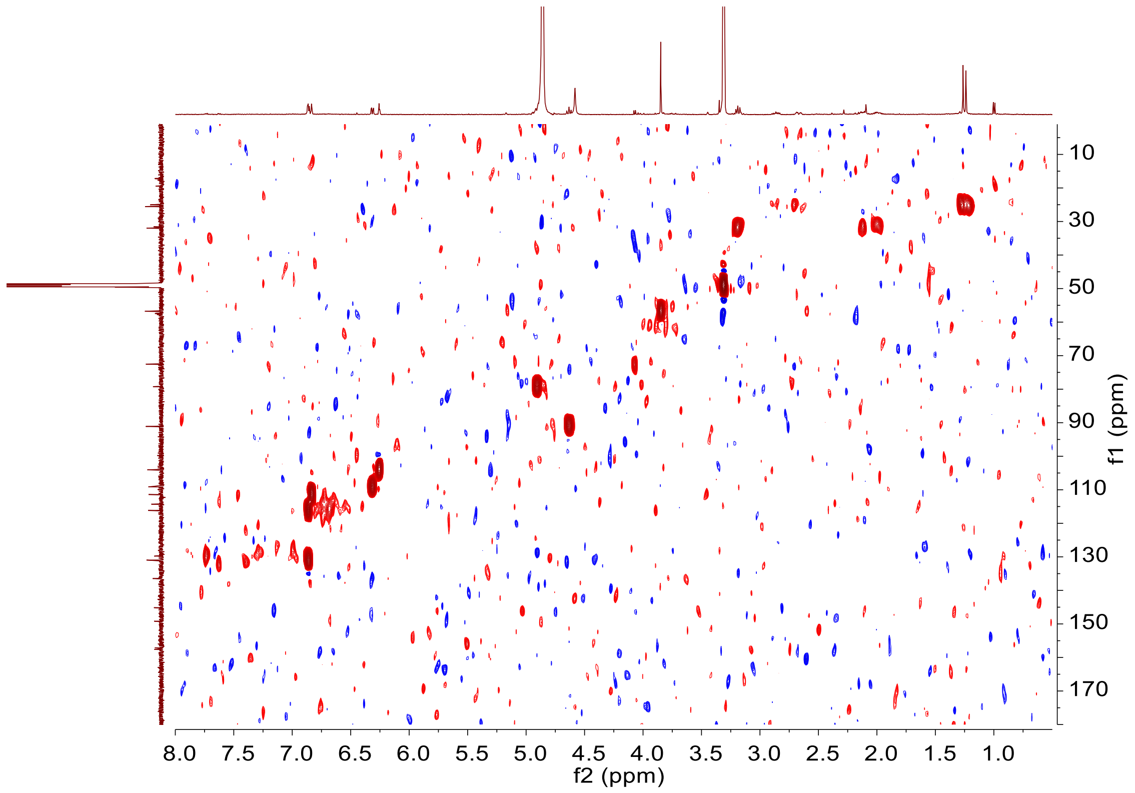

Figure S23: HSQC spectrum of **4** in methanol-*d*4

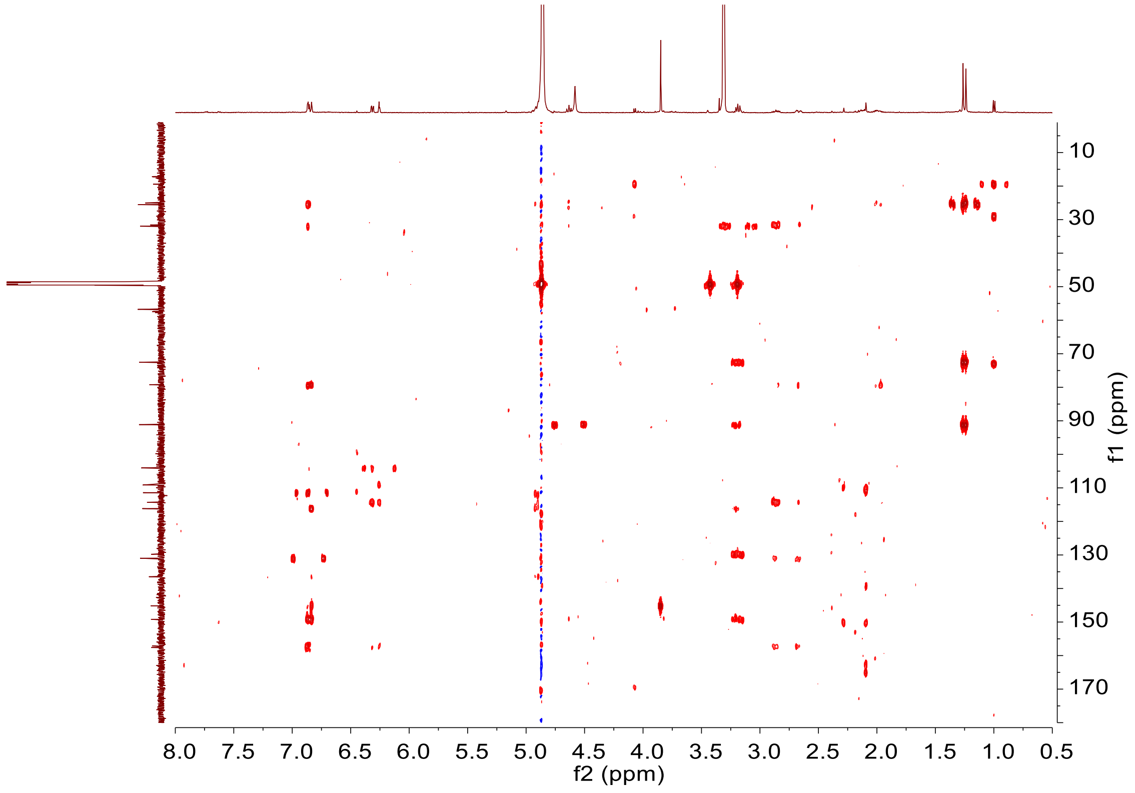

Figure S24: HMBC spectrum of **4** in methanol-*d*4

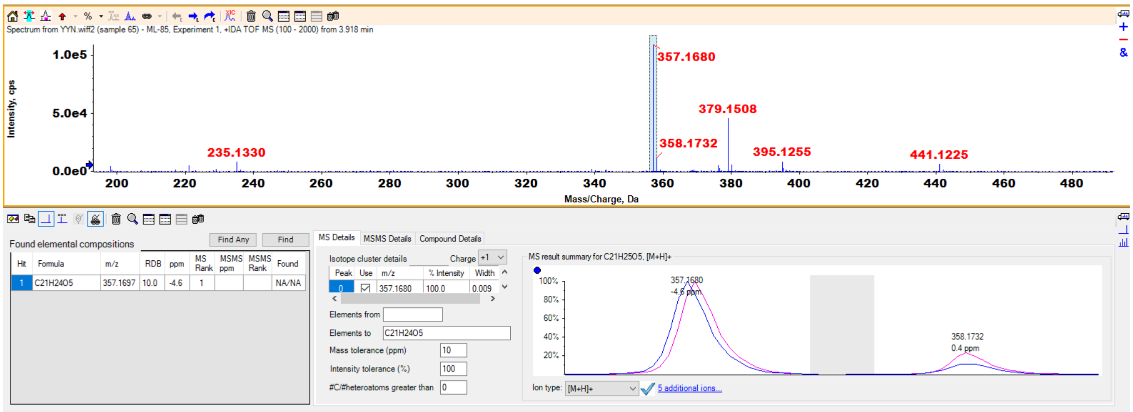

Figure S25: HRESIMS spectrum of **4**

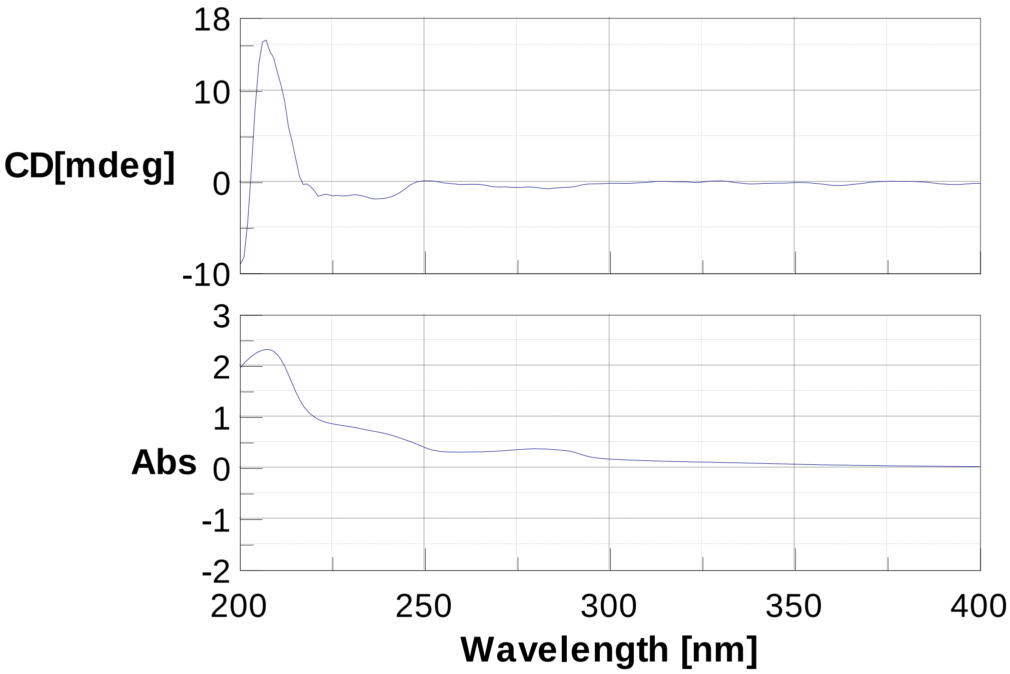

Figure S26: CD spectrum of **4** in MeOH

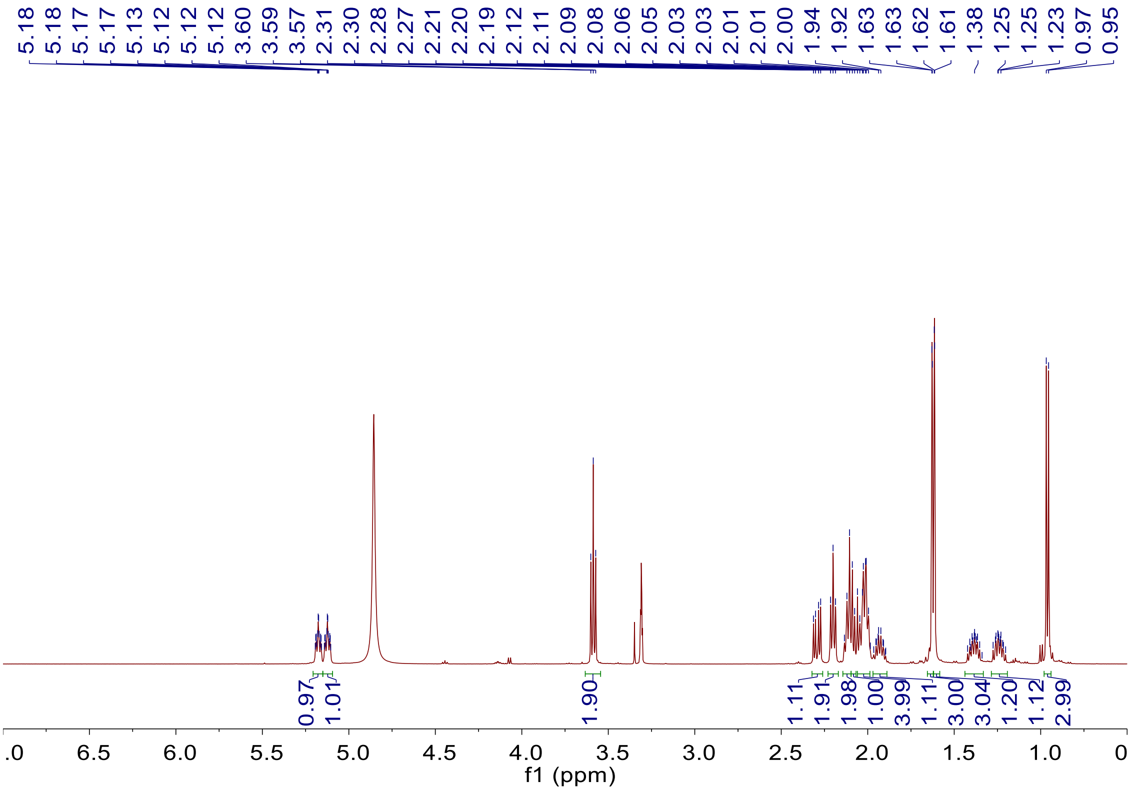

Figure S27: ^{1}H NMR spectrum of **7** in methanol-*d*4

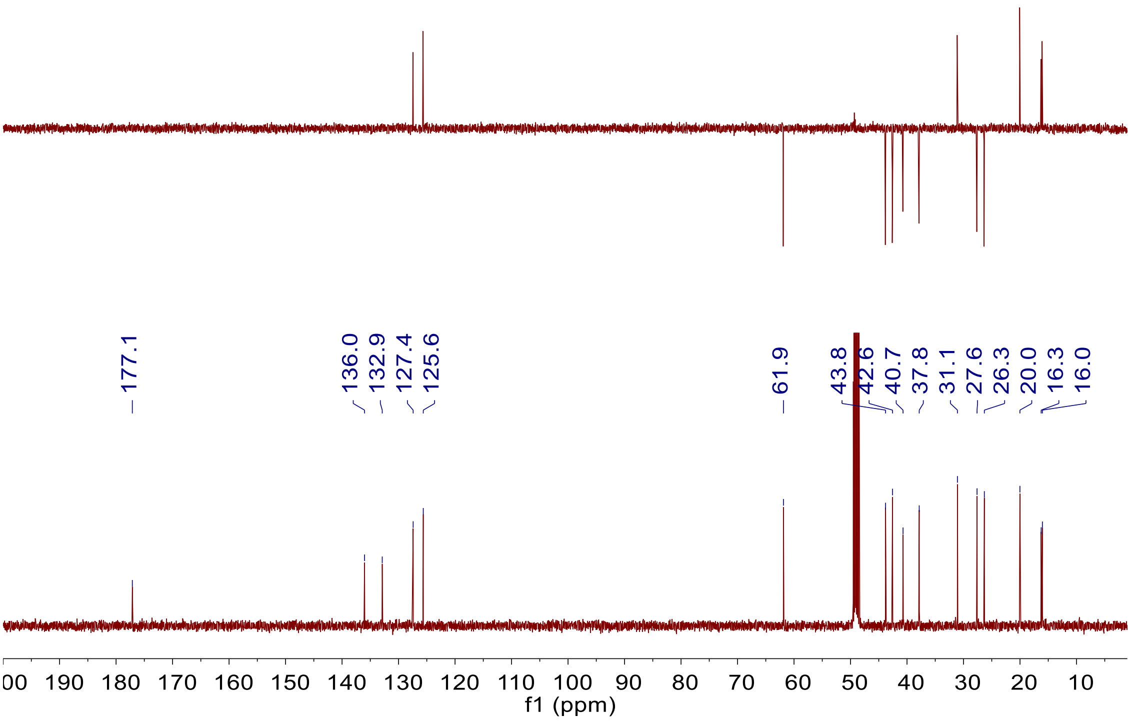

Figure S28: ^{13}C NMR and DEPT spectrum of **7** in methanol-*d*4

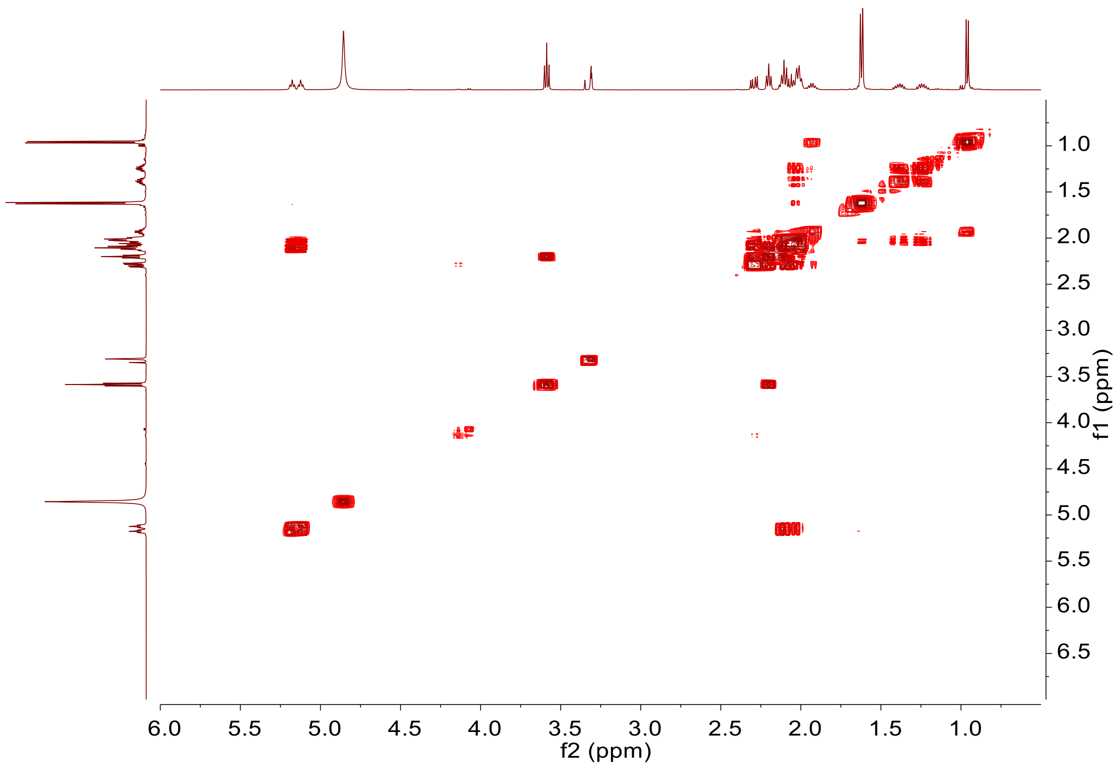

Figure S29: ^{1}H–^{1}H COSY spectrum of **7** in methanol-d_4

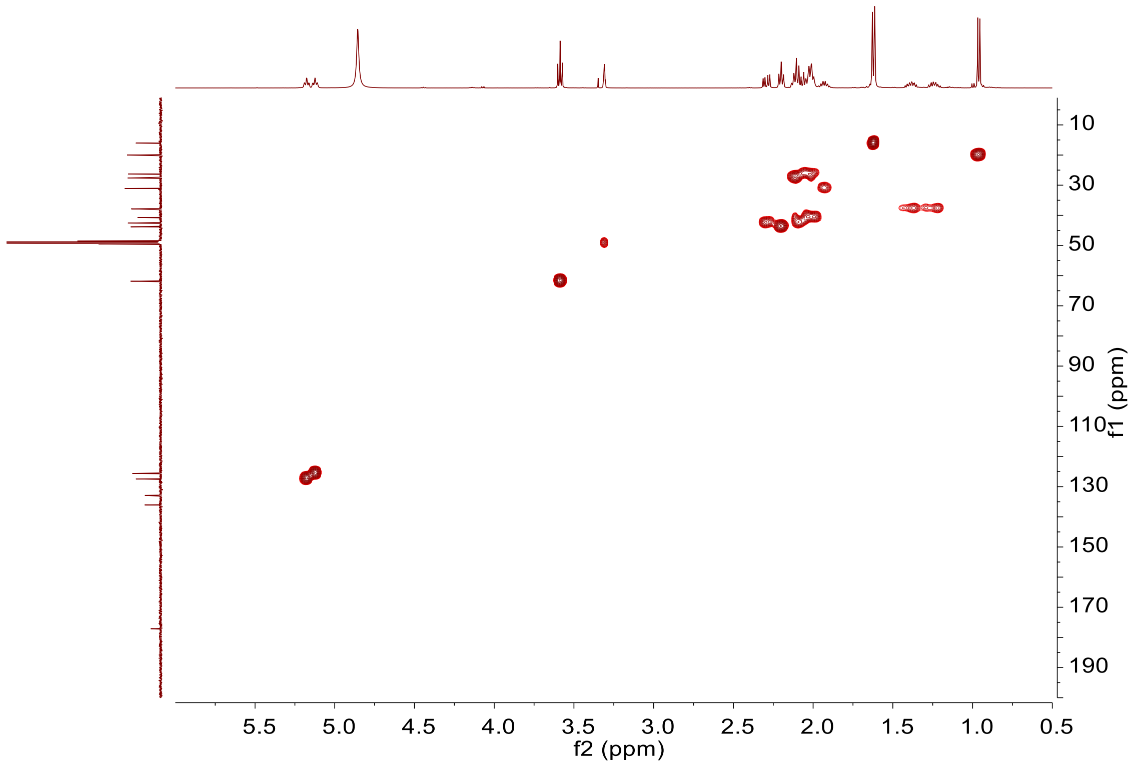

Figure S30: HSQC spectrum of **7** in methanol-d_4

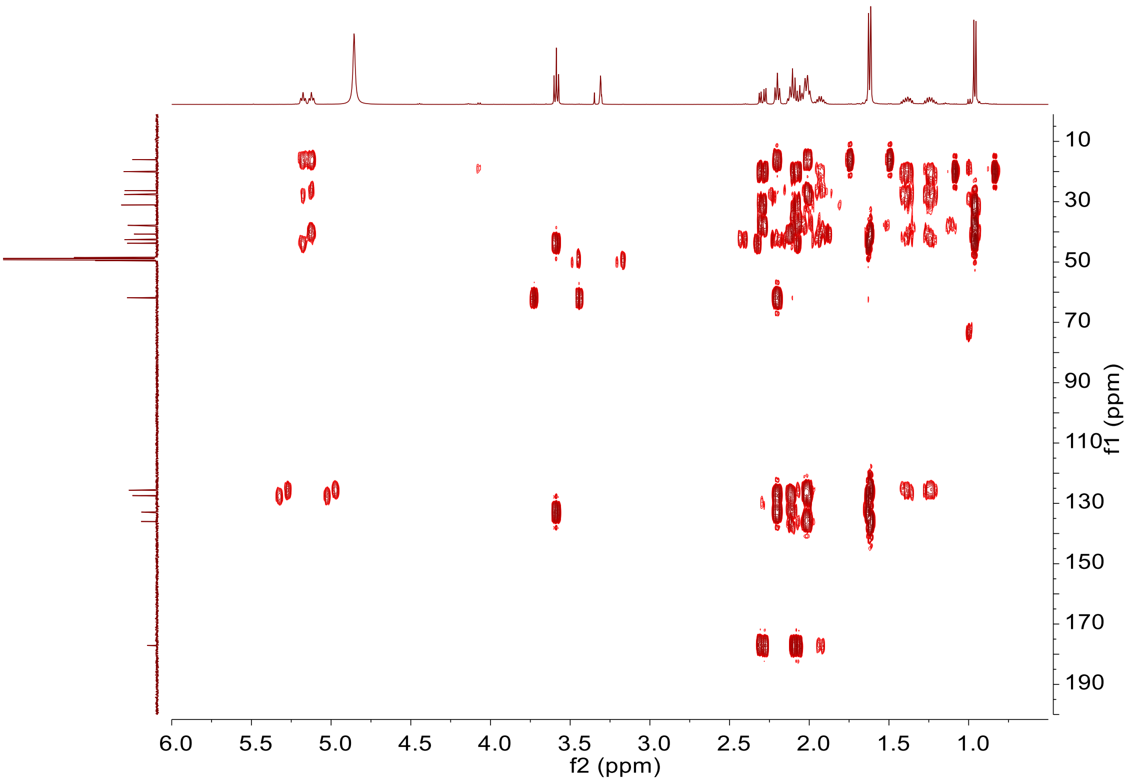

Figure S31: HMBC spectrum of **7** in methanol-*d*4

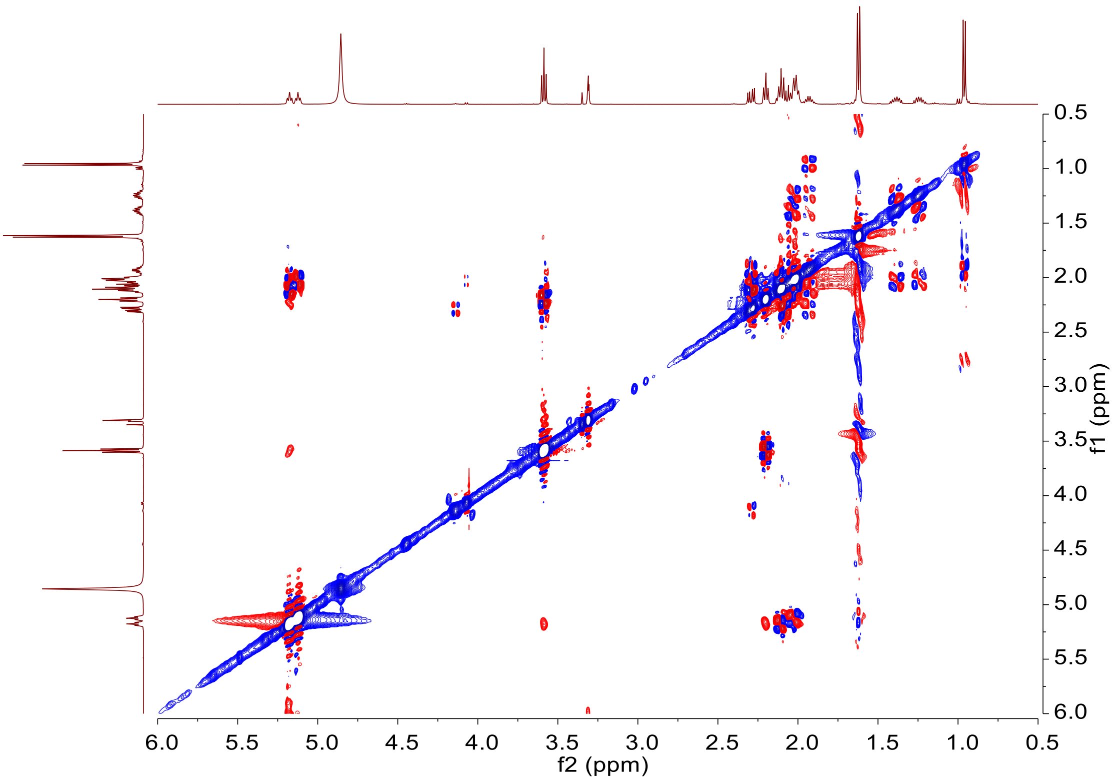

Figure S32: ROESY spectrum of **7** in methanol-*d*4

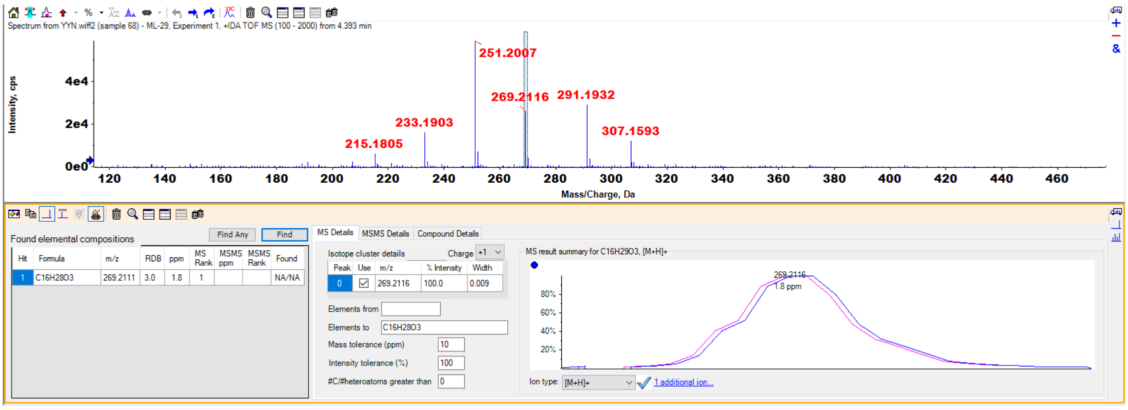

Figure S33: HRESIMS spectrum of **7**

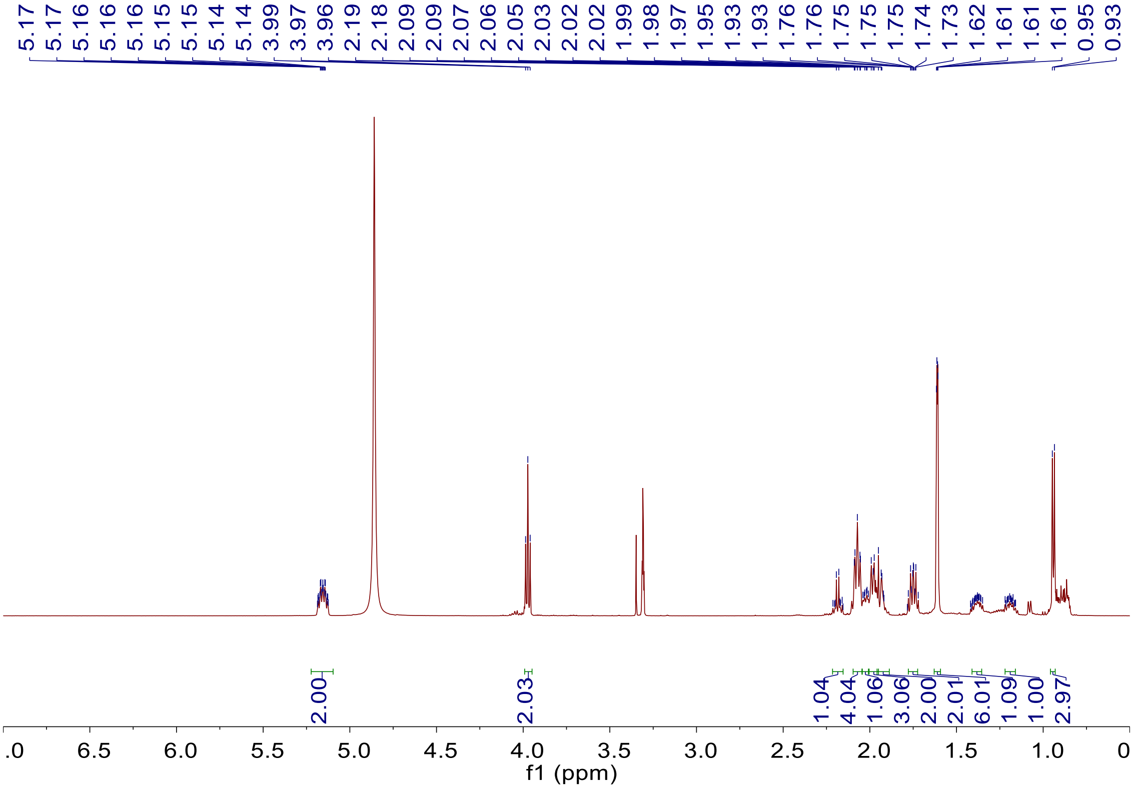

Figure S34: ^{1}H NMR spectrum of **8** in methanol-d_4

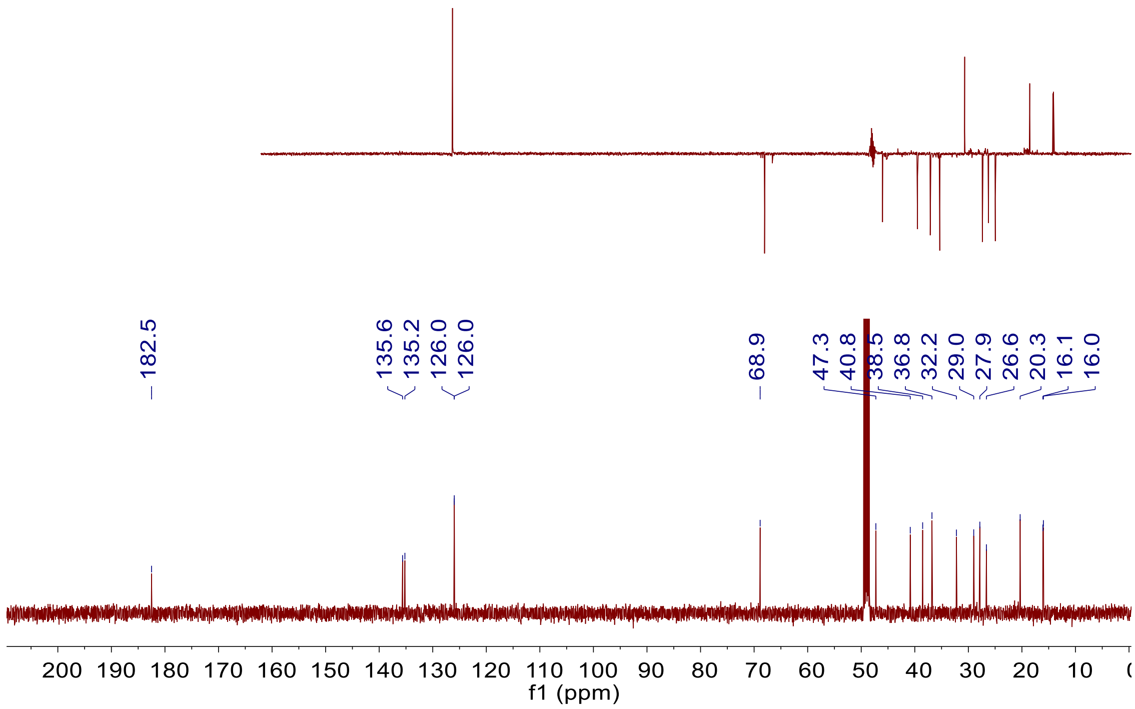

Figure S35: ^{13}C NMR and DEPT spectrum of **8** in methanol-*d*4

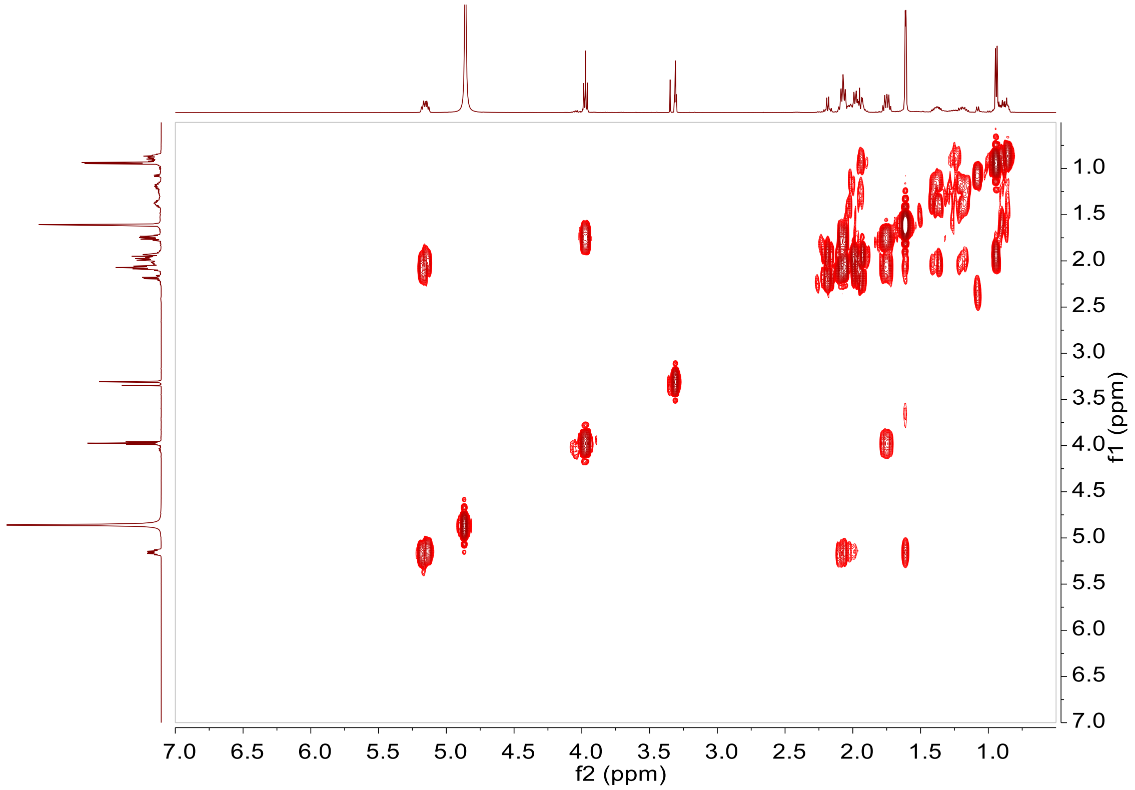

Figure S36: ^{1}H–^{1}H COSY spectrum of **8** in methanol-*d*4

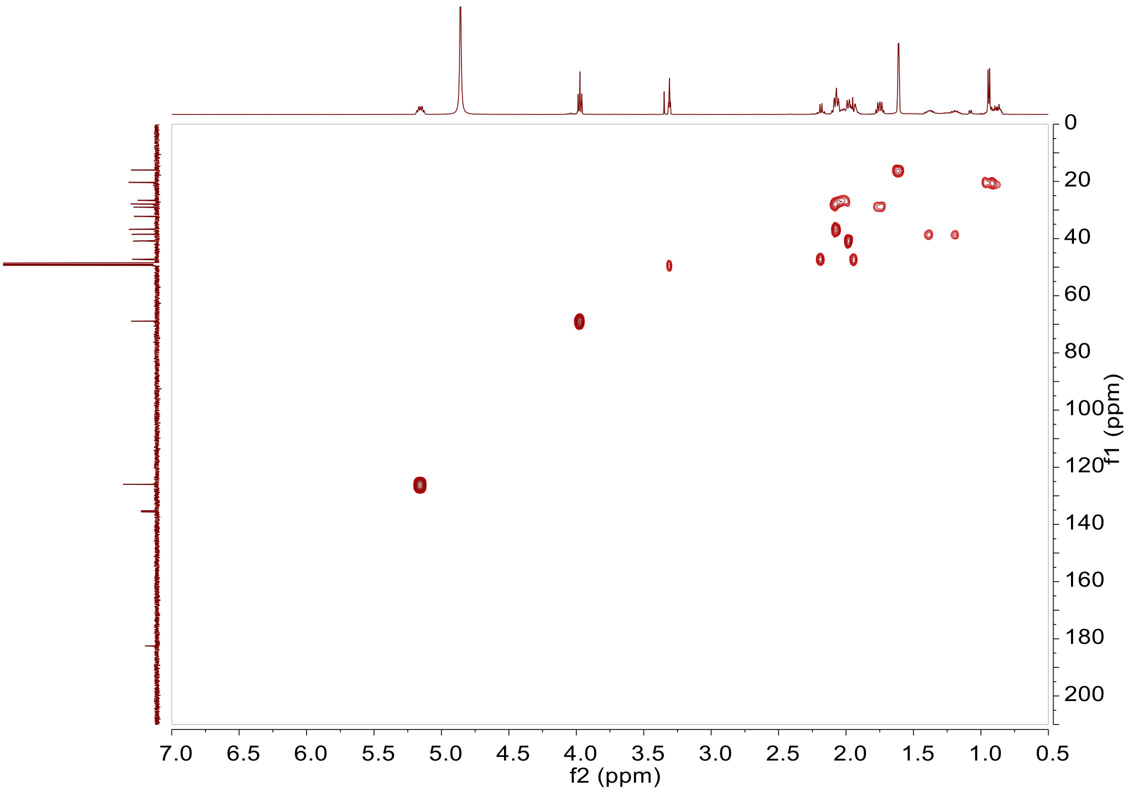

Figure S37: HSQC spectrum of **8** in methanol-*d*4

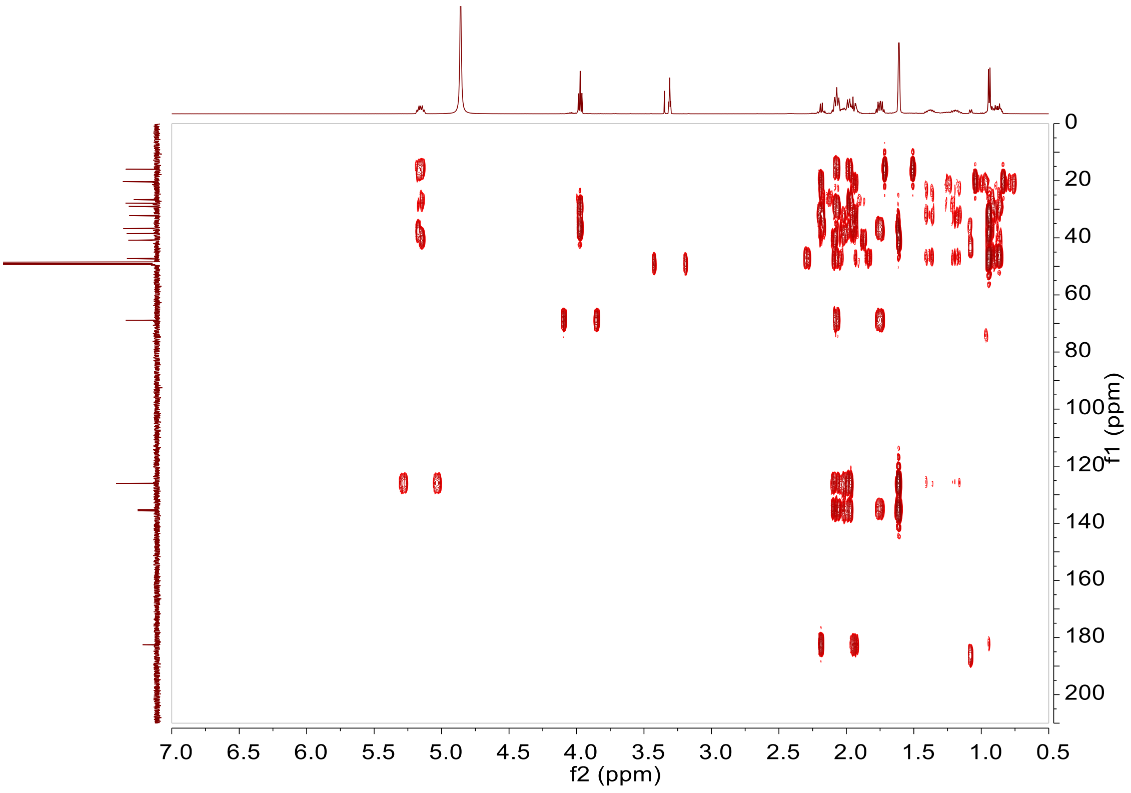

Figure S38: HMBC spectrum of **8** in methanol-*d*4

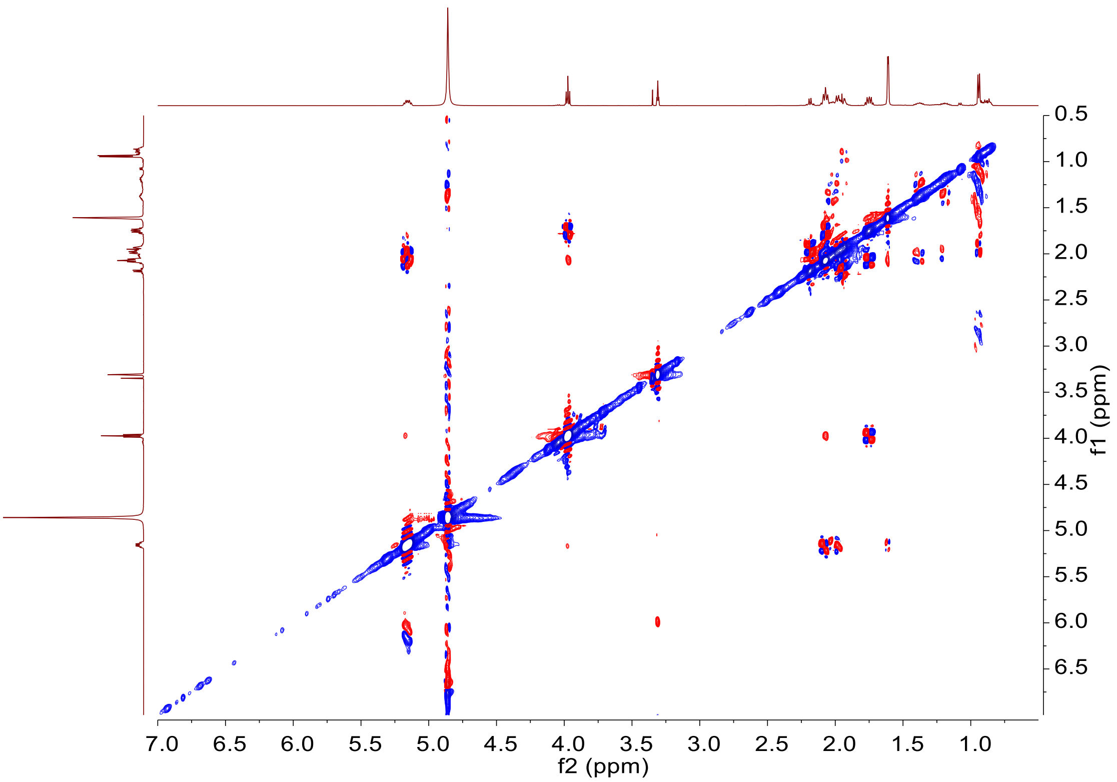

Figure S39: ROESY spectrum of **8** in methanol-d_4

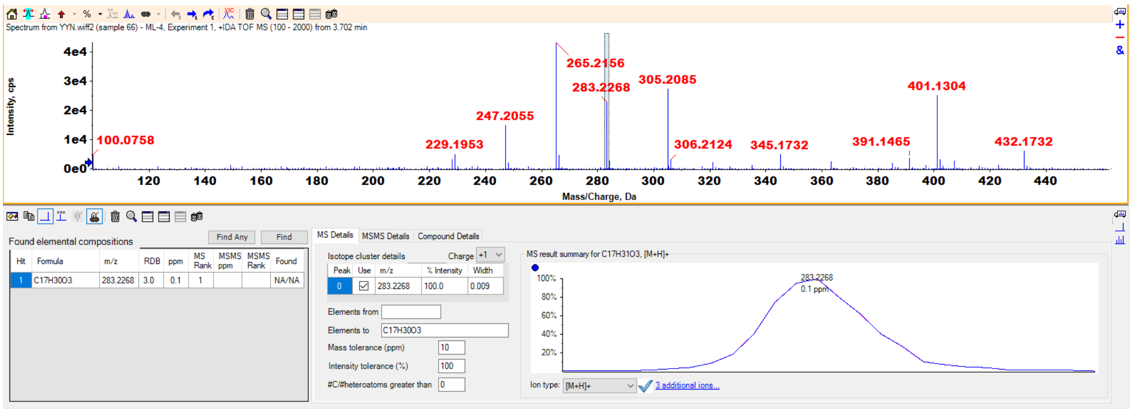

Figure S40: HRESIMS spectrum of **8**

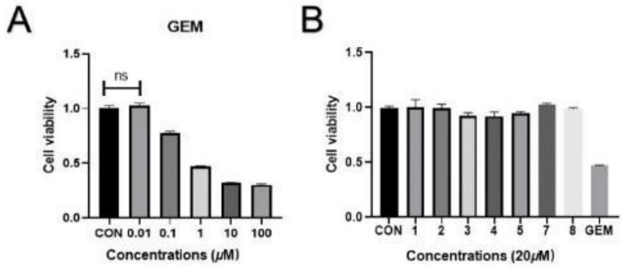

Figure S41. Compounds inhibited proliferation of Panc02-h7-GP-GFP cells. Cells were treated with gemcitabine, compounds at a 20 μM concentration, or DMSO for 48 h. The proliferation of Panc02-h7-GP-GFP cells in response to gemcitabine and the compounds was assessed by the CCK-8 assay. **A**, gemcitabine (GEM) inhibited proliferation of Panc02-h7-GP-GFP cells at 0.01, 0.1, 1, 10, and 100 μM concentrations. **B**, compounds were ineffective in inhibiting cell proliferation at 20 μM, using DMSO as a control and gemcitabine (GEM) at 1 μM as a positive control drug.

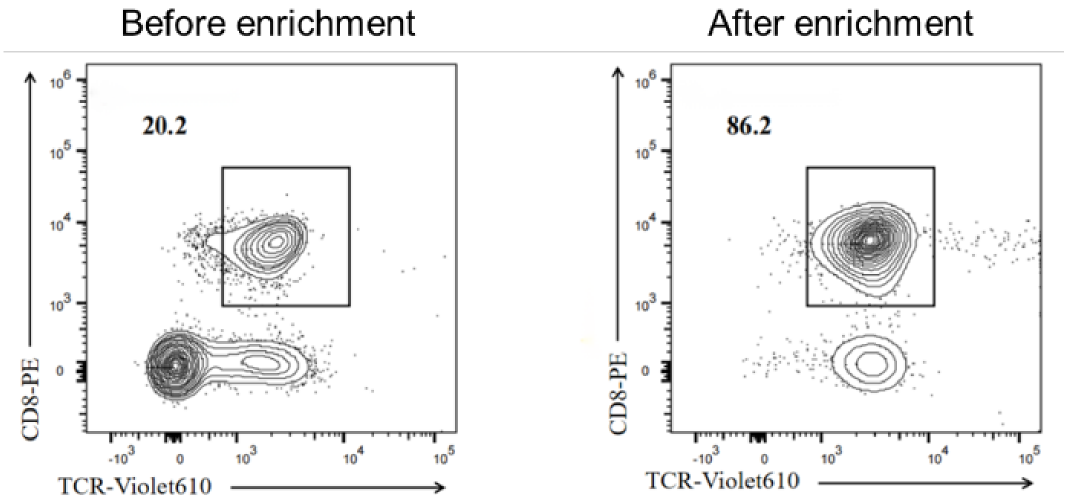

Figure S42: Purification of CD8$^+$T cells in vitro.

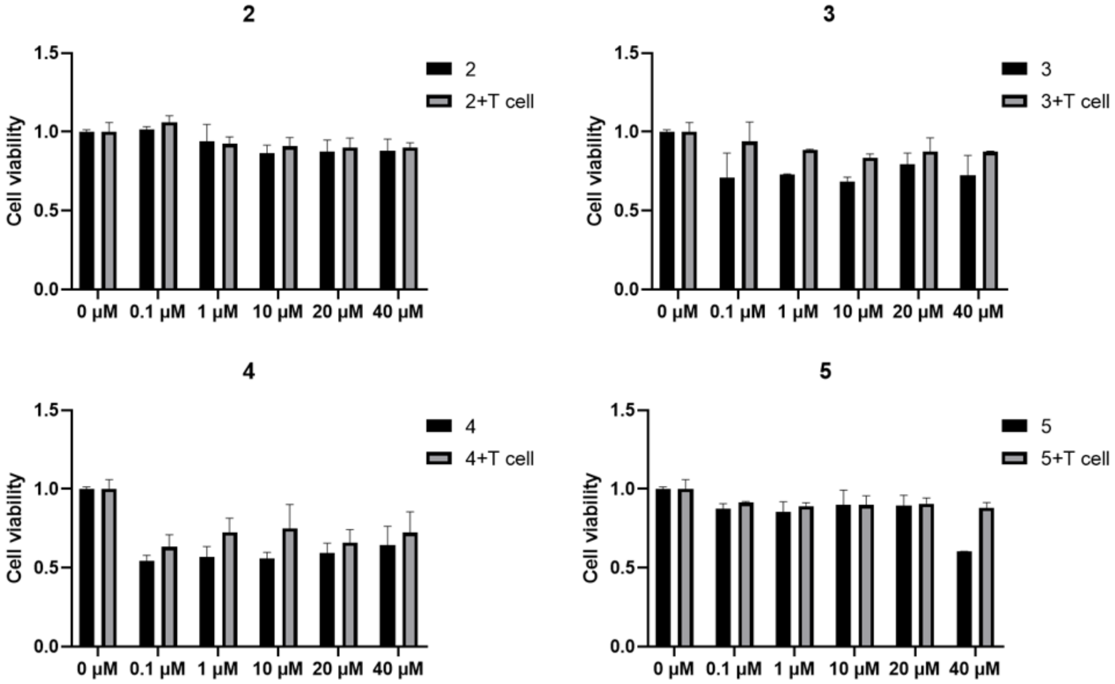

Figure S43: Compounds affected the antitumor activity of CD8⁺ T cells in vitro. Cells were incubated in the presence of the corresponding concentration of compounds or DMSO for 18 h and the fluorescence intensity was detected by a microplate reader (emission light 476 nm, excitation light 514 nm).
